# Allometry and Scaling of the Intraocular Pressure and Aqueous Humour Flow Rate in Vertebrate Eyes

**DOI:** 10.1371/journal.pone.0151490

**Published:** 2016-03-18

**Authors:** Moussa A. Zouache, Ian Eames, Amir Samsudin

**Affiliations:** 1 Institute of Ophthalmology, University College London, London, United Kingdom; 2 Department of Mechanical Engineering, University College London, London, United Kingdom; 3 University of Malaya, Kuala Lumpur, Malaysia; University of California San Diego, UNITED STATES

## Abstract

In vertebrates, intraocular pressure (IOP) is required to maintain the eye into a shape allowing it to function as an optical instrument. It is sustained by the balance between the production of aqueous humour by the ciliary body and the resistance to its outflow from the eye. Dysregulation of the IOP is often pathological to vision. High IOP may lead to glaucoma, which is in man the second most prevalent cause of blindness. Here, we examine the importance of the IOP and rate of formation of aqueous humour in the development of vertebrate eyes by performing allometric and scaling analyses of the forces acting on the eye during head movement and the energy demands of the cornea, and testing the predictions of the models against a list of measurements in vertebrates collated through a systematic review. We show that the IOP has a weak dependence on body mass, and that in order to maintain the focal length of the eye, it needs to be an order of magnitude greater than the pressure drop across the eye resulting from gravity or head movement. This constitutes an evolutionary constraint that is common to all vertebrates. In animals with cornea-based optics, this constraint also represents a condition to maintain visual acuity. Estimated IOPs were found to increase with the evolution of terrestrial animals. The rate of formation of aqueous humour was found to be adjusted to the metabolic requirements of the cornea, scaling as $V_{ac}^{0.67}$, where *V*_*ac*_ is the volume of the anterior chamber. The present work highlights an interdependence between IOP and aqueous flow rate crucial to ocular function that must be considered to understand the evolution of the dioptric apparatus. It should also be taken into consideration in the prevention and treatment of glaucoma.

## Introduction

Sight has been a key selective advantage in the evolution of species. The most elementary eyes evolved in early organisms more than 600 million years ago [1]. They involve at least one photoreceptor in the vicinity of shading pigment, and only allow for light detection [2]. More complex image-forming eyes evolved during the Cambrian explosion, around 540 million years ago [1]. They include an additional refractive element in front of the photoreceptor layer, which allow for increased light collection and enhanced optical performance [3]. While eyes fundamentally provide the same information about light intensity and wavelength [4], they occur in a variety of designs, shapes and sizes across animal phyla [3–5]. Morphological evidence suggests that eyes are polyphyletic and have evolved independently at least 40 times [6]. However, this is challenged by molecular experiments [7]. To date, the evolution of eyes remains largely subject to uncertainty [1, 3, 4].

**Fig 1:**
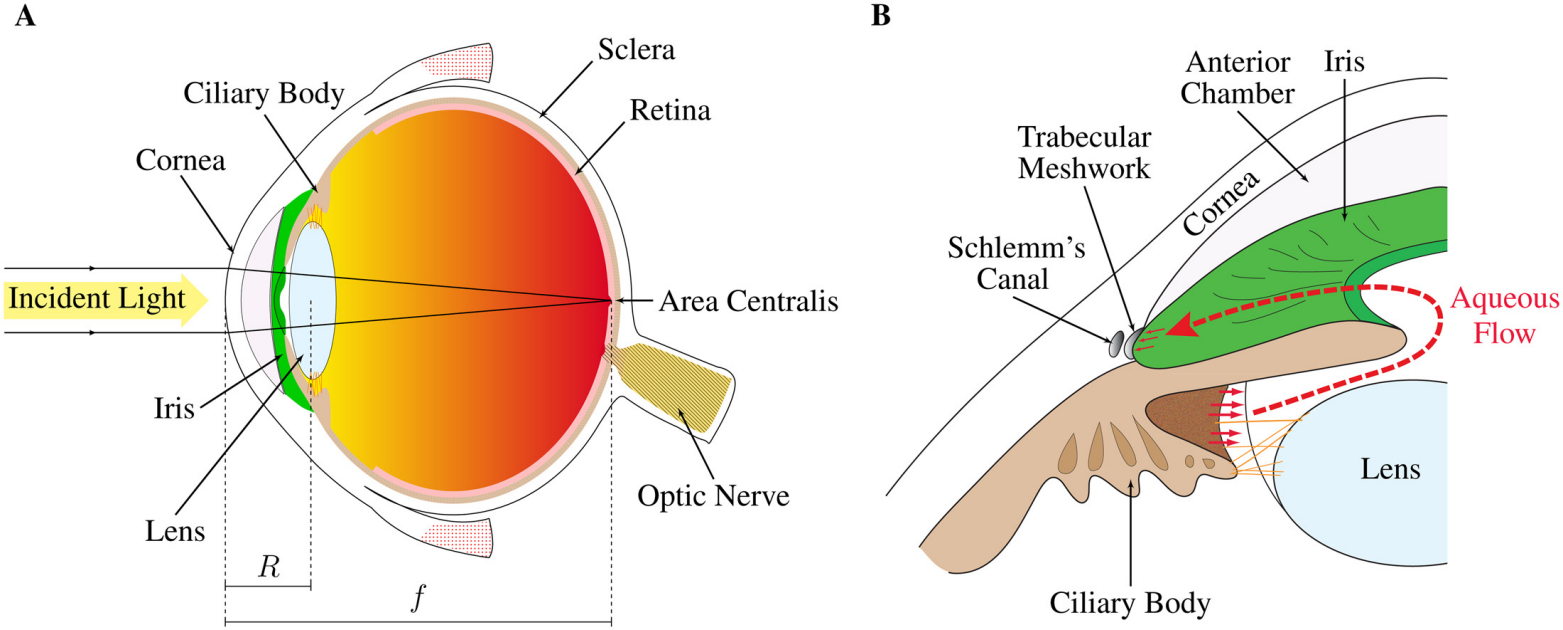
Schematic diagrams of a human eye (A) and the conventional aqueous flow pathway (B). The human eye (A), which is fairly representative of the vertebrate eye, is composed of concentric layers of tissue enclosing a fluid filled chamber. Light is scattered towards the back of the eye by the cornea and lens. Phototransduction is carried out in the retina. Most of the light is focused on an *area centralis*, which here coincides with the fovea. The intraocular pressure is maintained by the equilibrium between the formation of aqueous humour and the resistance to its outflow from the eye. Produced by the ciliary body, the aqueous humour flows around the iris into the anterior chamber and is drained through the trabecular meshwork and Schlemm’s canal (B).

Some of the most complex eyes are found among vertebrates. Vertebrate eyes are formed by concentric layers of tissue enclosing a fluid filled chamber (Fig 1A). Anteriorly, the dioptric apparatus responsible for scattering and directing light towards the photoreceptors is composed of the cornea and an epithelial lens [8]. The relative refractive power of the cornea and lens varies across vertebrates. In man, the cornea constitutes the main refractive element of the eye, while the lens (along with aqueous and vitreous humours) accounts for close to a third of the eye’s refractive power. It also contributes to accommodation for fine focusing [6]. In addition to its refractive function, the cornea can act as a protection for the eye, a light filter or a nutritive device [9–11].

In order to be used as optical instruments, it is crucial that vertebrate eyes remain turgid. This is achieved through the application of an intraocular pressure (IOP) on the internal surface of the eye cup. In man and various other vertebrates, the maintenance of an appropriate IOP is also important to preserve the curvature of the cornea, and therefore to retain visual accuracy [12]. In all vertebrates, the IOP is maintained by the equilibrium between the rate of formation of aqueous humour and the resistance to its drainage. Aqueous humour is produced by the ciliary body. It then flows around the iris to the trabecular meshwork and Schlemm’s canal (for primates) or angular aqueous plexus (for non-primates) (Fig 1B) [13]. The aqueous humour acts as a blood surrogate to the avascular cornea and lens, providing nutrition, regulating homeostasis and clearing metabolic wastes. As a result, in addition to maintaining the IOP, the inflow and outflow of aqueous humour are crucial to maintain a turnover rate sufficient to support the metabolic requirements of the dioptric apparatus [14]. In man, the trabecular meshwork and Schlemm’s canal form most of the entire resistance to aqueous humour outflow from the eye [14, 15].

Even though the set of elements composing the eye are common to all vertebrates, significant structural variations are observed across species. These variations are likely to be the result of an adaptation to differences in habitat or function rather than expressions of phylogenetic evolution [8]. Physical constraints common to all vertebrates are also likely to have played an important role in the formation of the ocular system of each species; however, they remain largely unexplored. Allometric and scaling analyses allow for the identification of such constraints, having for instance been used to predict the relationship between metabolic rate and body mass [16–20], between physiological rates, body size and life history traits [21] and between habitat loss and biodiversity [22]. In the eye, they have so far largely been limited to anatomical characteristics [23–26].

The relationship between IOP and the structure of the vertebrate eye has so far seen little investigation. It has been suggested that the IOP may be one of the factors regulating ocular growth at least in young mammals and birds [27, 28]. The aim of the present work is to examine the importance of the IOP and rate of formation of aqueous humour in the development of vertebrate eyes and to identify evolutionary constraints that are common to all vertebrates. In addition to underlining functional constraints that have shaped the vertebrate eye, the present work is important to build a better understanding of the dynamics of the IOP and aqueous humour flow rate. Dysregulation of the IOP is often pathological to ocular function. High IOP may cause damage to the optic nerve and lead to glaucoma, which is in man the second leading cause of blindness [29, 30]. In the present study, a series of models and scaling laws based on the functional importance of the dioptric apparatus are developed, and predictions from the model are compared with a dataset obtained through a systematic review.

## Analysis

### Relation between IOP and aqueous humour formation rate

The conventional aqueous flow pathway, represented in Fig 1B, includes an inflow at the ciliary body and an outflow through the trabecular meshwork and Schlemm’s canal or the angular aqueous plexus. Most of the aqueous humour ultimately leaves the eye through the episcleral vein. A small portion of the aqueous humour may enter the sclera and ultimately leave the eye either through the supraciliary space or the choroidal circulation. This outflow is commonly referred to as uveo-scleral outflow [31]. The steady balance between inflow and outflow of aqueous humour is described by Goldmann’s equation. By denoting Π the intraocular pressure (the notations used in this analysis are listed in Table 1), the rate of formation of aqueous humour *Q* satisfies

$$\begin{array}{c} Q=K\left(\Pi -P_{ev}\right)+F_{u}, \end{array}$$ (1)

where *K* is a bulk permeability, *P*_*ev*_ is the episcleral venous pressure and *F*_*u*_ represents the uveo-scleral outflow, which is taken to be insensitive to pressure variations [32, 33].

**Table 1:** List of notations used in the scaling and statistical analyses.

Parameter	Notation
Intraocular pressure	Π
Estimated Intraocular pressure	$\bar{\Pi }$
Aqueous humour flow rate	*Q*
Episcleral venous pressure	*P*_*ev*_
Ocular focal length	*f*
Radius of curvature of the cornea	*R*
Density of water	*ρ*
Density of the body of an animal	*ρ*_*b*_
Acceleration	*a*
Volume of the anterior chamber	*V*_*ac*_
Body mass	*M*
Time scale	*T*
Characteristic size	*L*
Characteristic speed	*U*
Flux of mechanical energy per unit area	*H*

### Scaling analysis

#### Variations in the focal length of the eye and corneal curvature during head movement

In the course of head movement, the eye is subject to a force that exerts a pressure on it and slightly distorts it. The IOP plays an important role in ensuring that the corneal curvature, here denoted *R*, and the focal length of the eye, here denoted *f*, remain relatively constant under this force. The focal length of the eye is largely related to the radius of curvature of the cornea *R*. The pressure change over the height or length of the eye due to acceleration scales as *ρaR*, where *ρ* is the density of water and *a* is the acceleration of the eye. The ratio of this pressure difference due to the IOP is *ρaR*/Π. When the radius of curvature of the cornea changes by *X*, *f* changes by a comparable amount. The presence of a vertical or horizontal pressure difference therefore causes the curvature of the cornea to change by a ratio

$$\begin{array}{c} X/R∼\rho aR/\Pi . \end{array}$$ (2)

#### Maintenance of the focal length of the eye and corneal curvature during movement

The focal length of the eye is impaired if it changes by a distance that scales with the size of the focal region. The size of the focal region *I*_*focal*_ scales with the size of the eye, and satisfies

$$\begin{array}{c} \Delta f<I_{focal}∼\lambda R, \end{array}$$ (3)

where λ ∼ 1/20. Thus the IOP must be sufficiently large to satisfy

$$\begin{array}{c} \rho aR/\Pi ∼X/R<\lambda , \end{array}$$ (4)

or

$$\begin{array}{c} \Pi >\rho aR/\lambda . \end{array}$$ (5)

This shows that the IOP is essentially an order of magnitude greater than the pressure drop across the eye due to acceleration caused by gravity or head movement.

#### Minimal IOP across vertebrates

Having arrived at Eq (5), the next step is to estimate how the product *aR* varies across species. The size of an animal approximately scales as *L* ∼ (*M*/*ρ*_*b*_)^1/3^, where *ρ*_*b*_ is the density of the body (typically, *ρ*_*b*_ is similar to the density of water)[34]. Associated with the movement of an animal is a characteristic time scale *T*. By denoting *U* = *L*/*T* the characteristic speed of the movement, the power associated with animal movement is *MaU*, which has dimensions *ML*^2^/*T*^3^. Mechanical energy is lost by radiation, heat flux through breathing or loss through the skin. Each of these losses occur over an area scaling as *L*^2^. Since the flux per unit area of mechanical energy *H* is similar in most vertebrates, the power associated with animal movement balances as

$$\begin{array}{c} ML^{2}/T^{3}∼L^{2}H. \end{array}$$ (6)

By substituting in the value of *M*, we have *L* ∼ *T*(*ρ*_*b*_/*H*)^1/3^, which means that the speed of the movement of an animal

$$\begin{array}{c} U=L/T∼{\left(\rho_{b}/H\right)}^{1/3} \end{array}$$ (7)

shows a weak dependence on the size of the animal, as supported by [35]. As a result,

$$\begin{array}{c} aR∼{\left(L/T\right)}^{2}∼{\left(\rho_{b}/H\right)}^{2/3} \end{array}$$ (8)

is also weakly dependent on the size of the animal. By combining Eqs (5) and (8), we obtain a criterion that the IOP must satisfy, namely

$$\begin{array}{c} \Pi >\frac{\rho }{\lambda }{\left(\frac{\rho_{b}}{H}\right)}^{2/3}. \end{array}$$ (9)

This inequality gives an estimate of the minimum value of the IOP across vertebrates. Since a high IOP may lead to structural and functional damage to the optic nerve, the constraint would typically be an equality constraint as it then conforms to the minimum pressure required for keeping the focal plane in place. In man, the typical IOP may be estimated by substituting in typical values for the acceleration of the eye occurring during walking, running or just movement of the head. Since the typical vertical acceleration of the head is 0.3−0.5 × *g* [36], which scales as *g*, the IOP must satisfy:

$$\begin{array}{lll} \Pi >\rho gR_{human}/\lambda & ∼ & \left(10^{3}\text{kg}/{\text{m}}^{3}\right)\times \left(10\text{m}/{\text{s}}^{2}\right)\times \left(0.01\text{m}\right)\times 20\\ & ∼ & 2000\text{Pa}=15\text{mmHg}. \end{array}$$

**Fig 2:**
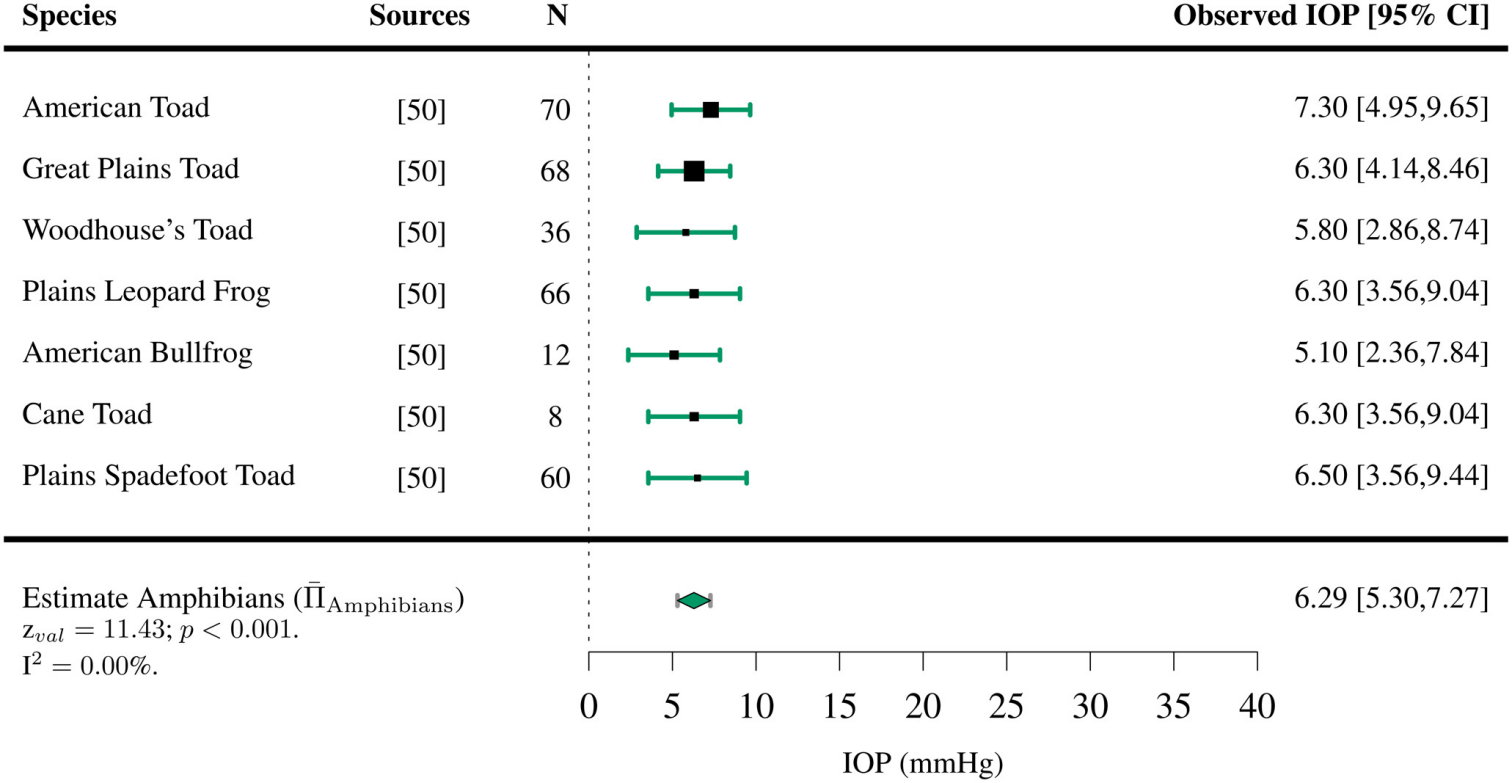
Estimated intraocular pressure ($\bar{\Pi }$) in amphibians. IOPs were collected through a systematic review (S1 Table and Table A in S1 File). The studies the data was extracted from, the sample size *N*, the observed IOP and the 95% confidence interval (CI) are indicated for each species.

This value is within the range of the IOP measured in healthy human eyes (Fig 2E).

#### Scaling of the rate of formation of aqueous humour

In addition to maintaining the IOP, the formation of aqueous humour supports the metabolic requirements of the cornea and lens. Corneas are typically formed of a few noncellular and cellular layers, for which aqueous humour constitutes the primary supply of oxygen and glucose [37]. Compared with the cornea, the lens has a low metabolic rate [38, 39] so that the contribution of the aqueous humour to lens metabolism is negligible. Therefore, it is expected that the rate of formation of aqueous humour is proportional to the area of the cornea. Since the area of the cornea scales as $V_{ac}^{2/3}$, where *V*_*ac*_ is the volume of the anterior chamber, the aqueous humour flow is expected to scale as

$$\begin{array}{c} Q∼V_{ac}^{0.67}. \end{array}$$ (10)

### Systematic review

The objective of the systematic review was to determine typical IOPs, rates of formation of aqueous humour and volumes of the anterior chamber among healthy adult vertebrates.

#### Data sources

Searches were undertaken on PubMed, Scopus and BioOne databases from their inception to 31st August 2015. A list of journals specialised in veterinary ophthalmology was established and their respective databases of articles were searched manually. The terms ‘intraocular’, ‘intraocular pressure’, ‘tonometry’, ‘aqueous humour’, ‘ophthalmic examination’ and ‘ocular parameters’ were searched individually and then combined with ‘species’, ‘vertebrate’, ‘amphibian’, ‘bird’, ‘mammal’, ‘fish’, ‘reptile’, ‘terrestrial’ and ‘aquatic’. Reference lists were hand-searched and citations of articles of interest were screened. Conference abstracts were included. Additionally, a list of books on veterinary ophthalmology and comparative physiology of the eye was established and each of them was searched manually.

#### Inclusion criteria

Since the IOP varies significantly between juvenile and adult animals [40–43], studies reporting IOP only for juvenile animals were excluded. If IOPs were reported separately for juvenile and adult animals, only values for the adult group were considered. Studies were eligible for inclusion if they reported more than one measurement, the mean and standard deviation, 95% confidence interval (CI) or median and range of the IOP in adult animals regardless of their sex or the eye (left or right) in which the measurement was taken. Studies where the size of the sample was not reported were excluded. Studies reporting IOPs in genetically modified animal strains were excluded. Studies reporting the rate of formation of aqueous humour were only included if they also specified the mean volume of the anterior chamber in the group of animals sampled. Studies designed to test the effect of pharmacological compounds on IOP and/or rate of formation of aqueous humour were excluded unless they included an untested control group. In this case, only measurements carried out on the control group were extracted. For each species, the typical body mass was extracted either from the studies reporting the IOP or the rate of formation of aqueous humour or from published and unpublished sources.

#### Data abstraction

Data was collected with a customised data extraction form (Tables A–E in S1 File). In cases where median and range were reported, mean and standard deviation were infered using published formulas [44]. Within each studies, if more than one IOP was reported for the same group, mean and standard deviations were combined. For each species, if more than one study reported IOPs, rate of formation of aqueous humour or volume of the anterior chamber then the weighted mean and weighted standard deviation were computed.

### Statistical analysis and testing of predictions from the scaling analysis

#### Characteristics of the IOP among vertebrates

The statistical analysis was carried out using the software R [45]. A main outcome measure of the systematic review was the raw mean IOP in the five classes of vertebrates: amphibians, birds, fish, mammals and reptiles. IOPs in the different classes of vertebrates were estimated by pooling the IOP in the different species using a random-effect model [46]. Forest plots were used to visualise the IOP among the different classes of vertebrates and within the whole dataset. The I^2^ statistic was used to assess the heterogeneity of the IOP within each class of vertebrate and across the whole dataset. The effect of vertebrate classes on the heterogeneity of the IOP was investigated by including the animal classes in the model as moderators. The significance of the effect was assessed by performing a Wald-type *χ*^2^-test.

#### Weak dependence between IOP and body mass

From Eq (9), the IOP is expected to have a weak dependence on body mass. This was tested by performing a non-parametric hypothesis test for pairwise statistical correlation between IOP and body mass based on Kendall’s rank correlation (*τ*) accounting for ties [47]. Kendall’s rank correlation was computed separately for each group of vertebrates and for the whole dataset. The rank correlation was considered statistically significant at *p* ≤ 0.05.

#### Scaling of the rate of formation of aqueous humour

In order to test Eq (10), rates of formation of aqueous humour were fitted to a power-law of the form $Q=\alpha V_{ac}^{\beta }$, where *α* and *β* are two dependent variables. The error structure of the dataset was determined by calculating the relative likelihood of multiplicative and additive errors. This was achieved by computing the values of a second order variant of Akaike’s information criterion (AIC_*c*_), which corrects for small sample size [48, 49]. The goodness of the fit was assessed by computing the coefficient of determination *r*^2^.

## Results

### Characteristics of the IOP among vertebrates

**Fig 3:**
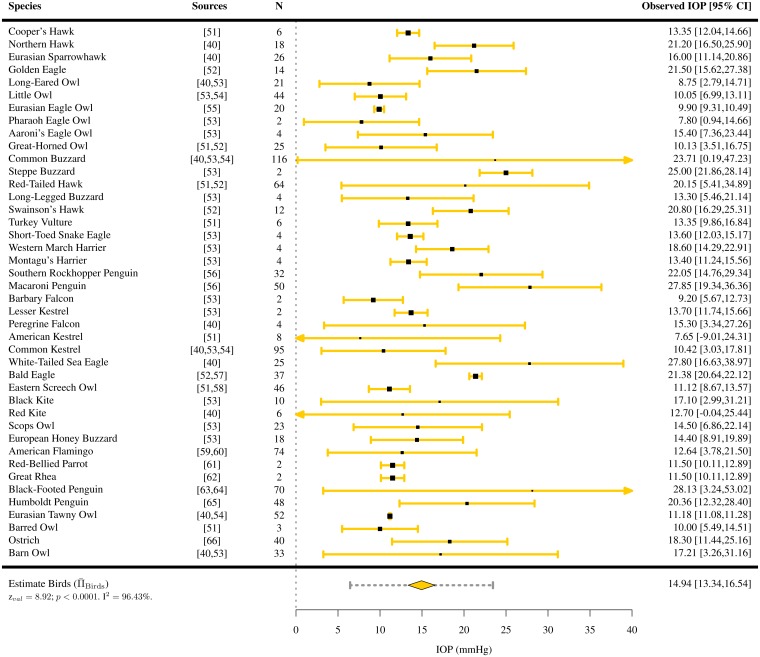
Estimated intraocular pressure ($\bar{\Pi }$) in birds. IOPs were collected through a systematic review (S2 Table and Table B in S1 File). The studies the data was extracted from, the sample size *N*, the observed IOP and the 95% confidence interval (CI) are indicated for each species.

**Fig 4:**
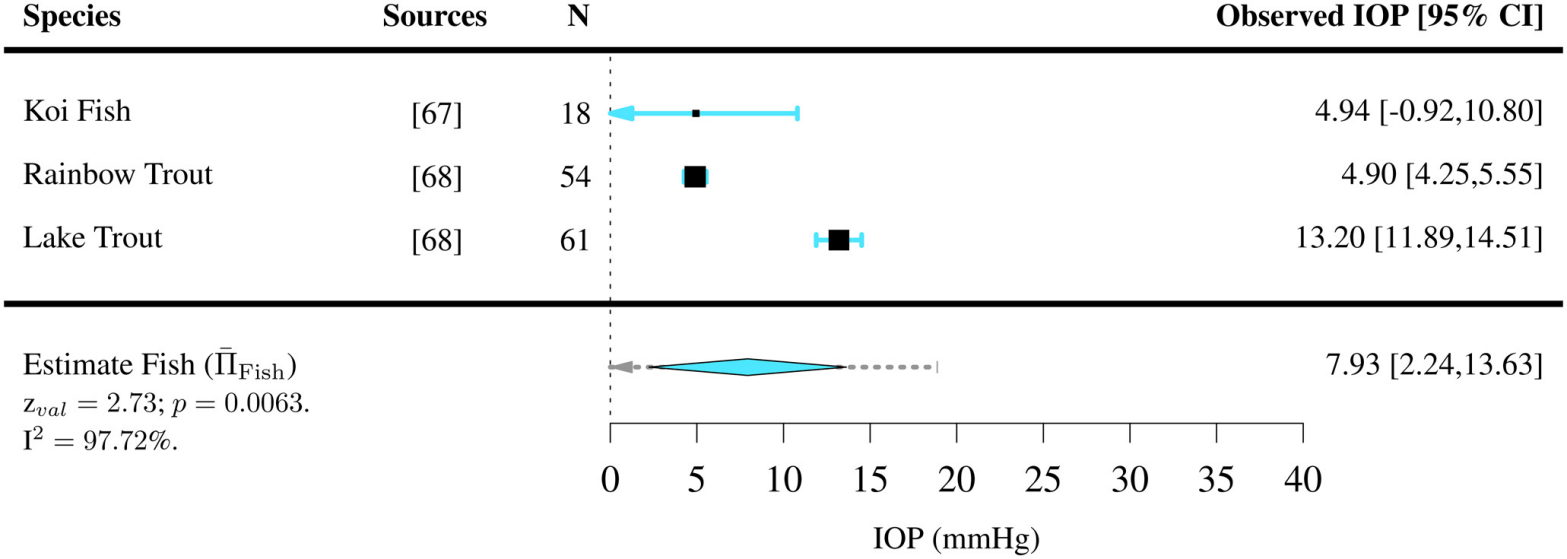
Estimated intraocular pressure ($\bar{\Pi }$) in fish. IOPs were collected through a systematic review (S3 Table and Table C in S1 File). The studies the data was extracted from, the sample size *N*, the observed IOP and the 95% confidence interval (CI) are indicated for each species.

**Fig 5:**
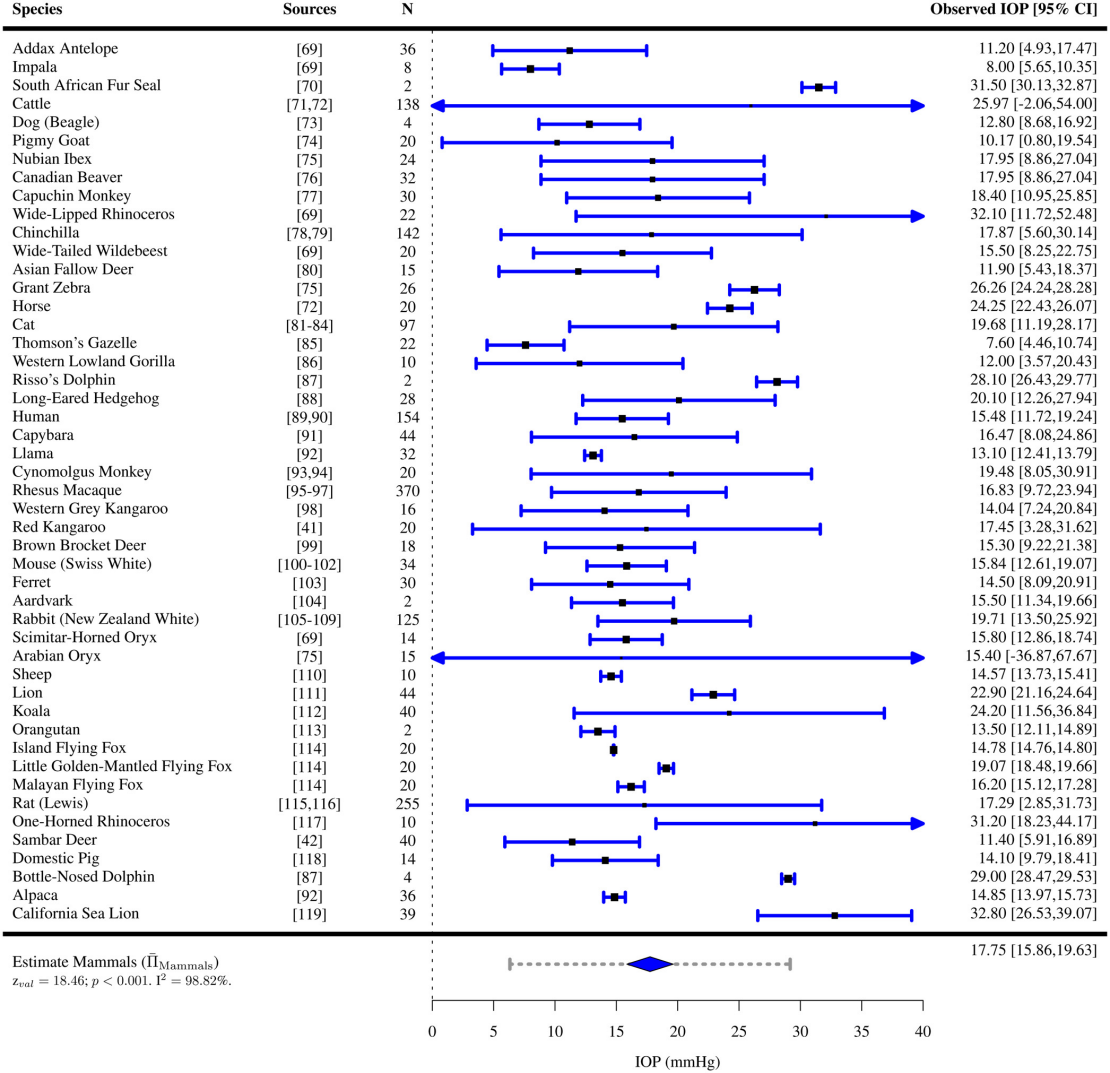
Estimated intraocular pressure ($\bar{\Pi }$) in mammals. IOPs were collected through a systematic review (S4 Table and Table D in S1 File). The studies the data was extracted from, the sample size *N*, the observed IOP and the 95% confidence interval (CI) are indicated for each species.

**Fig 6:**
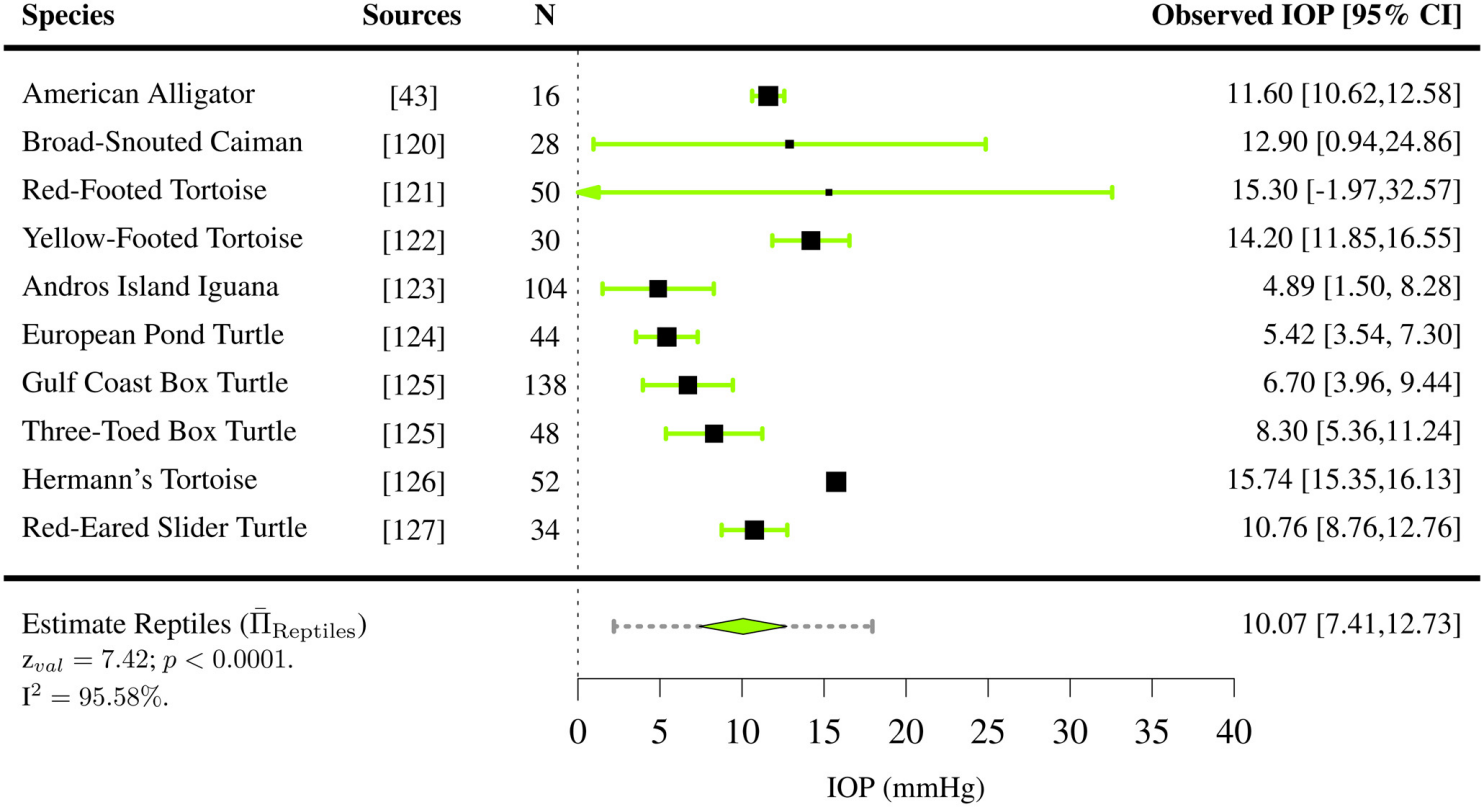
Estimated intraocular pressure ($\bar{\Pi }$) in reptiles. IOPs were collected through a systematic review (S5 Table and Table E in S1 File). The studies the data was extracted from, the sample size *N*, the observed IOP and the 95% confidence interval (CI) are indicated for each species.

**Fig 7:**
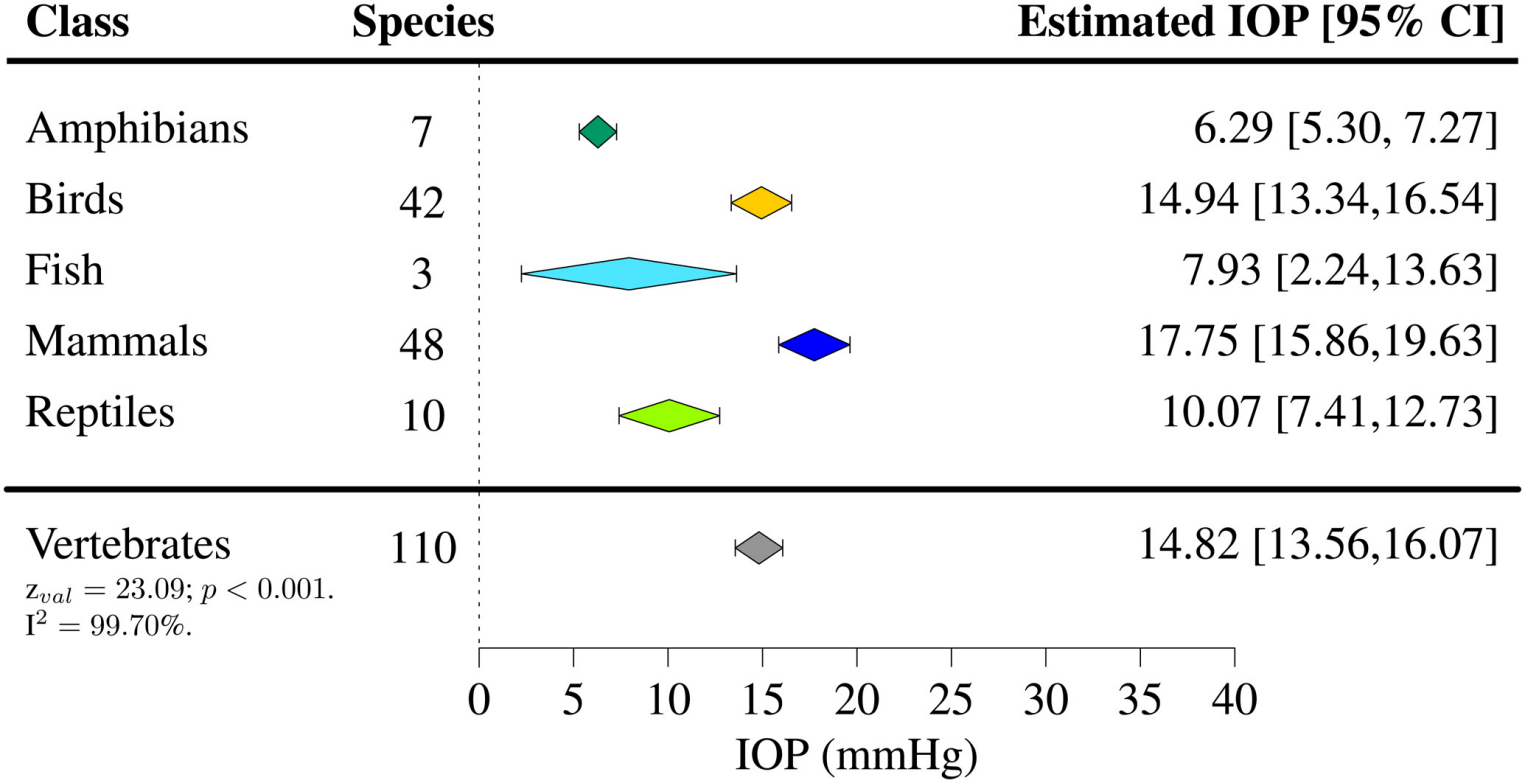
Estimated intraocular pressure ($\bar{\Pi }$) across vertebrates. IOPs were collected through a systematic review (S1–S5 Tables and Tables A–E in S1 File). The number of species included in the study, the estimated IOP and the 95% confidence interval (CI) are indicated for each class of vertebrates.

IOPs were extracted for 110 species of vertebrates (7 species of amphibians [50], 42 species of birds [40, 51–66], 3 species of fish [67, 68], 48 species of mammals [41, 42, 69–119] and 10 species of reptiles [43, 120–127]). For the majority of them, only one study per species satisfied the inclusion criteria. Among all vertebrates present in the study, the mean IOP was found to lie between 4.89 and 32.8 mmHg (Figs 2–6). The lowest estimated IOP was found among amphibians (${\bar{\Pi }}_{\text{Amphibians}}=$ 6.29 mmHg, Fig 2). The estimated IOPs of fish and reptiles were found to be respectively ${\bar{\Pi }}_{\text{Fish}}$ = 7.93 mmHg and ${\bar{\Pi }}_{\text{Reptiles}}=$ 10.07 mmHg (Figs 4 and 6). The estimated IOP among birds and mammals were the largest (${\bar{\Pi }}_{\text{Birds}}$ = 14.94 and ${\bar{\Pi }}_{\text{Mammals}}$ = 17.75 mmHg respectively) (Figs 3 and 5). The characteristics of the IOP in each class of vertebrates are summarised in Fig 7.

A high level of heterogeneity was found in all classes of vertebrates (I^2^ > 95%, with I^2^ = 99.70% across vertebrates) apart from amphibians. Including the different classes of vertebrates as moderators to the model accounted for 33.76% of the heterogeneity in effect (*p* < 0.001).

### Dependence of the IOP to body mass

**Fig 8:**
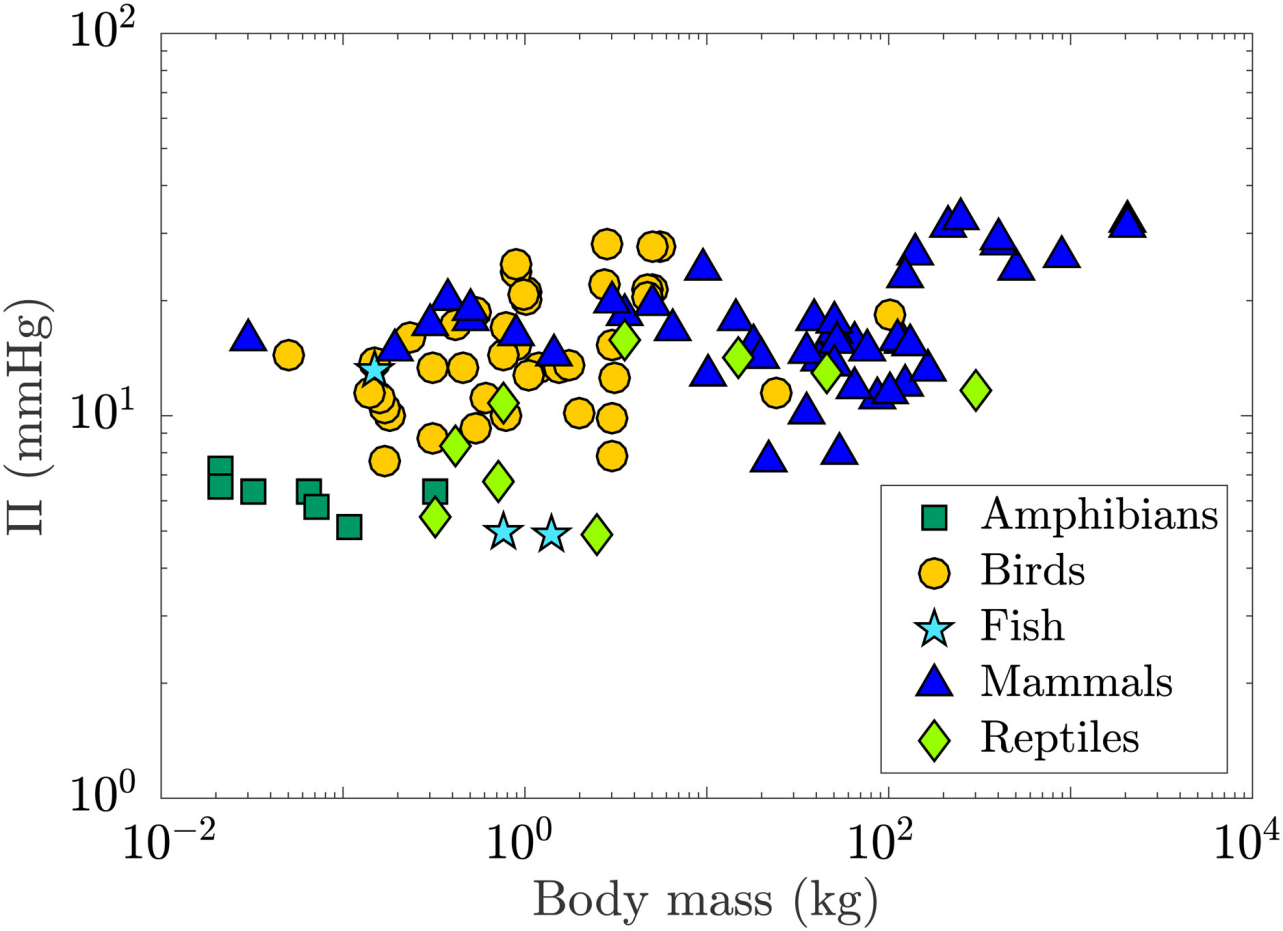
Log-log plot representing the evolution of the IOP (Π) with typical body mass in vertebrates. The IOP was found to correlate weakly with body mass across all vertebrates (*τ* = 0.296, *p* < 0.001).

Among the species present in the study the typical body mass varied over 6 orders of magnitude (from Plains Spadefoot Toad to Rhinoceros) while the typical mean IOP was found to lie between 4.89 and 32.8 mmHg (Fig 8). The rank correlation between IOP and body mass was found to be weak in birds and mammals (*τ* = 0.26, *p* = 0.015 and *τ* = 0.12, *p* = 0.23 respectively) and moderate in reptiles (*τ* = 0.39, *p* = 0.39); however, the effect was statistically significant in birds only (Table 2). In amphibians, IOP and body mass were found to be strongly negatively correlated (*τ* = −0.62); however, the effect was not statistically significant (*p* = 0.085). For all vertebrates, a weak statistical dependence between the IOP and body mass was found (*τ* = 0.296, *p* < 0.001). This is in agreement with Eq (9), which shows that the IOP has a weak dependence on body mass among vertebrates.

**Table 2:** Kendall’s rank correlation (*τ*) and 2-sided *p*-value for IOP and body mass among vertebrates. Kendall’s rank correlation could not be computed for fish because typical IOPs were found in three species only.

	*τ*	2-sided *p*-value
**Amphibians**	-0.62	0.085
**Birds**	0.263	0.015
**Fish**	–	–
**Mammals**	0.12	0.23
**Reptiles**	0.389	0.18
**All vertebrates**	0.296	5.36 ×10^−6^

### Scaling of the aqueous humour flow rate

**Fig 9:**
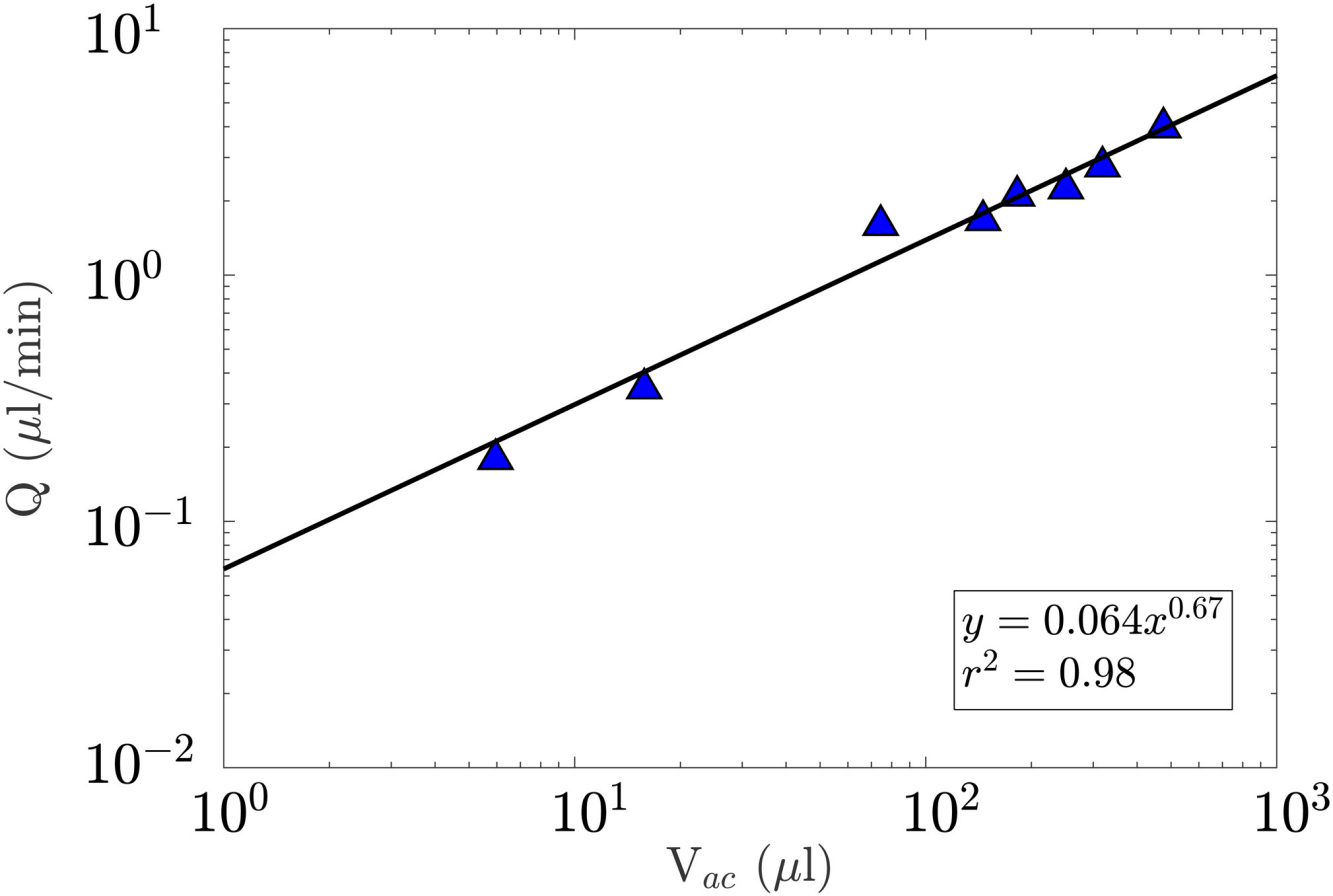
Log-Log plot representing the evolution of the rate of formation of aqueous humour (*Q*) with the volume of the anterior chamber (*V*_*ac*_) in various vertebrates. A fit (plain line) was obtained through model averaging. The rate of formation of aqueous humour was found to scale as $Q∼{\text{V}}_{ac}^{0.67}$ , which is in agreement with scaling Eq (10).

Nine studies satisfied the inclusion criteria of the systematic review. Apart from human where two studies were used, the mean rate of formation of aqueous humour and the mean volume of the anterior chamber were extracted in only one study per species (Table 3). Since there was similar support for additive normal and multiplicative log-normal error structures (AIC_*c*_ = 9.59 and 8.50 respectively), the value of the dependent variables *α* and *β* were obtained through model averaging [49]. This yielded *α* = 0.064 (95% CI [0.046,0.26]) and *β* = 0.67 (95% CI [0.41,0.73]), which is in support of Eq (10). The fit is plotted on a logarithmic scale in Fig 9.

**Table 3:** Means of the rate of formation of aqueous humour (*Q*) and the volume of the anterior chamber (*V*_*ac*_) with respective standard deviations in various vertebrates. The systematic review yielded measurements of *Q* and *V*_*ac*_ in eight species only, all of them mammals. Only one study per species (apart from human) satisfied the inclusion criteria.

Species	Common name	Sources	Sample size (eyes)	Mean *Q* (*μ*l.min^−1^)	Standard Deviation (*μ*l.min^−1^)	Mean *V*_*ac*_(*μ*l)	Standard Deviation (*μ*l)
*Aotus trivirgatus*	**Owl Monkey**	[128]	16	2.75	0.46	317	67
*Felis Catus*	**Cat**	[81]	15	3.98	0.05	479	*n.i.*
*Homo sapiens sapiens*	**Human**	[89, 90]	154	2.08	0.009	183.36	84.21
*Macaca fascicularis*	**Cynomolgus Monkey**	[129]	7	1.6	3.66	74	*n.i.*
*Macaca mulatta*	**Rhesus Macaque**	[97]	24	1.68	0.02	144.54	12.82
*Mus musculus*	**Mouse (Swiss White)**	[100]	8	0.18	0.05	5.9	0.5
*Oryctolagus cuniculus*	**Rabbit (New Zealand White)**	[130]	4	2.25	0.56	250	*n.i.*
*Rattus norvegicus*	**Rat (Lewis)**	[116]	10	0.35	0.11	15.65	3.3

## Discussion

In this paper, a scaling analysis of the IOP and the rate of formation of aqueous humour were carried out in order to identify evolutionary constraints common to all vertebrate eyes and further the understanding of the physiology of the dioptric apparatus. Predictions from the scaling analysis were tested using IOPs and rates of formation of aqueous humour from a pool of vertebrates collected through a systematic review. It was shown that in order to maintain the focal length of the eye and the curvature of the cornea during head or body movement, the IOP needs to be an order of magnitude greater than the pressure drop across the eye that results from movement. An estimate of the minimal value of the IOP among vertebrate was determined, and it was shown that the IOP is weakly dependent on body mass. An allometric analysis of the rate of formation of aqueous humour showed that it scaled to $V_{ac}^{0.67}$. This was in good agreement with data collected through the systematic review.

### Quality of the data collected through systematic review

The analysis carried out in the present work constitutes the first attempt at characterising variations in the IOP and aqueous flow rate among vertebrates. One limitation pertains to the quality and consistency of the data collected from the literature, which is contingent on the methods used to make measurements. In most studies the IOP was assessed using commercially available tonometers, which vary by the type of tonometry that they are based on and their respective readings. Rebound tonometry was proven to provide significantly different measurements when compared to applanation tonometry [58, 127]. Statistically significant differences in readings from two of the most commonly used tonometers (TonoVet^®^ and TonoPen XL^®^) have also been reported [55, 56, 60]. Furthermore, some tonometers require an animal-specific calibration from the manufacturer, which may not be available at the time of measurement. Since the method used to measure the IOP is likely to be affected by the size of the eye and the thickness and curvature of the cornea [131], this may lead to incorrect measurements.

The aim of the systematic review was to collect typical IOPs for healthy adult species of vertebrates; therefore, some aspects of the variations in IOP within each group of vertebrates were not included in the study. While in healthy animals the IOP is generally not significantly different in the left and right eye [40, 41, 43, 53, 56–59, 64–66, 69, 74–76, 79, 85, 88, 91, 92, 98, 103, 111, 114, 114, 117, 122–127, 132] or in males and females [41, 43, 56, 58, 63, 65, 69, 74, 75, 79, 85, 88, 91, 92, 103, 114, 121–124, 126, 132, 133], it has been found to change with position in flamingos and bats. [59, 60, 114]. These variations are also present in man, and have been associated with changes in episcleral pressure [134, 135]. The IOP was also found to change with age in some species [41, 56, 63]. While no statistical correlation between IOP and body weight was found in raptors [53], Long-Eared Hedgehog [88] and juvenile Yacare Caiman [133], a moderate statistically significant negative correlation was found in Gulf-Coast Box Turtles and Three-Toed Turtles [125].

Measurements of the rate of formation of aqueous humour are sparse as compared to the IOP, and are mostly restricted to laboratory animals. This is not surprising as it involves measuring the flushing rate of a dye from the eye, and therefore requires prolonged and sometimes difficult animal handling. The number of values used in the present analysis could have been increased by including studies that did not report the mean volume of the anterior chamber in the group of animals where aqueous humour flow rate was measured. However, this inclusion criteria was important as the volume of the anterior chamber varies greatly not only between animals, but also between genetically modified strains of the same species [136].

Since the evaluation of the IOP is used to diagnose glaucoma in animals, the data collected in the present study is of important value to veterinary ophthalmologists. Although in man the IOP is routinely measured in the clinics, its evaluation in animals is not systematic. Reference IOPs have yet to be produced for several species, and it is often necessary to compare measurements in an animal to other species from a similar group to diagnose pathologies.

### Limitations of the statistical analysis

The statistical analysis of the data collected through the systematic review was carried out to support the scaling analysis, which formulated fundamental functional constraints applicable to all vertebrates. A limitation of the statistical analysis pertains to the small number of values found for amphibians, fish and reptiles as compared to birds and mammals. More measurements over a broader range of animals would make the estimated IOPs more representative within each animal group. This is particularly true of fish as only three species were present in the study.

An underlying assumption of the statistical analysis used to test Eqs (9) and (10) is that the IOP, the body weight, the rate of formation of aqueous humour and the volume of the anterior chamber in each species are independent. This assumption is challenged by the fact that species share an evolutionary history [137]. Despite being a non-parametric test [138], the significance of Kendall’s rank correlation may be affected by the phylogenetic relationship that animals share. The regression between rate of formation of aqueous humour and volume of the anterior chamber assumes that both variables follow a bivariate distribution, which may not be realistic in the context of evolution.

The fact that the IOP of closely related vertebrates such as Impalas and Thompson’s gazelle (*Antilopinae* subfamily, both IOPs below 8mmHg), Scimitar-Horned Oryx and the Wildebeest (*Hioppotraginae* subfamily, both IOPs close to 15 mmHg) and Horse and Zebra (*Equidae* family, both IOPs above 24 mmHg) lies within a similar range has led some to hypothesise that the IOP has phylogenetical similarities. However, the IOP of the closely related Llama and Alpacas (*Camelidae* family) and Scimitar-Horned Oryx and Arabian Oryx lie within different ranges [69]. Furthermore, the IOP among genetically modified strains of zebrafish vary by an order of magnitude [139], while significant differences in the volume of the anterior chamber have been observed in two genetically modified mice [136]. While the present work offers valuable functional explanations for some aspects of the shape of the vertebrate, future statistical analysis taking into account the phylogeny of species of vertebrates [140] would further strengthen our conclusions and clarify the phylogenetic component of the IOP.

### Evolutionary constraints associated with the IOP

The present analysis shows that the value of the IOP needs to be adjusted in order to maintain the focal length of the eye during movement. This constitutes an evolutionary constraint that applies to all vertebrates. As such, it has played an important role in shaping the structure of vertebrate eye in all the ecosystems that it adapted to. To the best of our knowledge, this evolutionary constraints has never been emphasised before.

In most terrestrial species, the cornea constitutes the main refractive element of the eye. The light scattered and directed towards the retina by the dioptric apparatus needs to be focused on the focal region of the eye for clear vision [6]. In many species, this focal region coincides with an area of the retina having distinctive anatomical characteristics such as the highest concentration of cones. This area takes various shapes in different animals, forming either a “bandlike” area called a visual streak or a circular area called *area centralis* (some birds have two *area centralis*, one of them located in the periphery of the eye for improved peripheral vision) [141]. In man, the *area centralis*, highlighted in Fig 1A, coincides with the fovea, and is about 200–400 *μ*m in diameter [142].

In species relying predominantly on the cornea to control the focusing of light onto the focal region (typically in terrestrial species), scalings Eqs (5) and (9) represent conditions that must be satisfied in order to maintain visual accuracy, and therefore constitute a second evolutionary constraint associated with the IOP. It is important to stress that this constraint is contingent on the relative importance of the lens in vision, which varies among animals. Even though in species with lens-based optics (such as fish) the effectiveness of the dioptric apparatus requires the maintenance of the focal length of the eye, clear vision is mostly determined by the design of the lens [6, 8, 143].

### Evolutionary constraints associated with aqueous humour flow

The rate of formation of aqueous humour was expected to scale as $V_{ac}^{0.67}$. This was found to be in good agreement with data extracted from the literature. Controversy remains over the accuracy and fundamental basis of metabolic scaling laws. Kleiber’s law suggests that basal metabolic rate scales to *M*^3/4^, where *M* is the body mass [16, 17]. Although it has shown good agreement in many studies [19, 144], some have argued that the basal metabolic rate rather scales to *M*^2/3^[18, 20, 145]. Others have suggested that the relationship between mass and metabolic rate is not a pure power law [146]. Perhaps remarkably, the metabolic supply to the dioptric apparatus does not have the same fractal-based supply limitations that have been proposed as an explanation for general physiological scaling [147]. The scaling of the aqueous humour flow rate to the metabolic requirement of the cornea suggests that alternative structural constraints are at play in the maintenance of the dioptric apparatus.

In the context of the evolution of the vertebrate eye, given the importance of aqueous humour dynamics in the maintenance of the IOP, scaling Eq (10) points towards a fine tuning between the volume of the anterior chamber, the surface area of the cornea and the turnover rate and resistance to outflow of aqueous humour. This constitutes a fundamental evolutionary constraint that is likely to have shaped the dioptric apparatus in all vertebrates. The present work shows that variations in the surface area of the cornea, which may arise from functional adaptations to certain ecosystems, require modifications of the rate of formation of aqueous humour (which may affect the structure of the ciliary body) and therefore possibly of the anatomy and physiology of the trabecular meshwork and Schlemm’s canal/angular aqueous plexus. It may also necessitate adjustments of other structures of the eye to possible changes in the IOP.

More measurements in a wider range of vertebrates would provide additional support for the analysis carried out here and further the understanding of the tuning between IOP, *Q* and *V*_*ac*_.

### IOP in the evolution of the vertebrate eye

**Fig 10:**
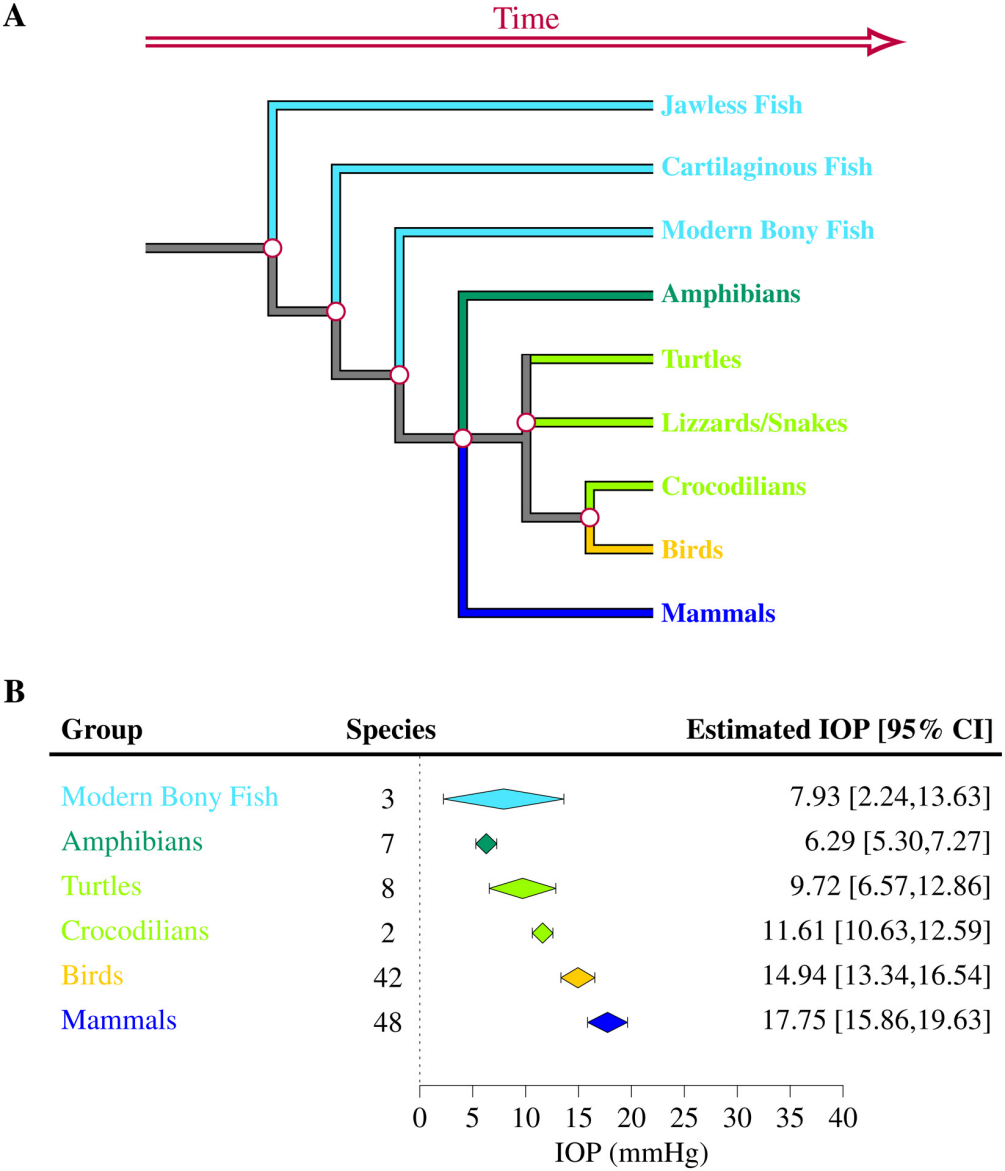
Simplified phylogenic tree of the vertebrate family (A) and estimated IOP within each group (B). The diagram was not plotted to scale. The sample size *N* and the 95% confidence interval (CI) is indicated for each group. The estimated IOP appears to have increased with the evolution of terrestrial animals.

From the aspect of the structure of the eye, vertebrates can be divided into three different great groups: the *ichthyopsida* composed of fish and amphibians, the *sauropsida* formed by reptiles and birds and the *mammalia*. The vertebrate eye initially evolved for vision in shallow water. It has over time adapted itself for vision in a wide range of habitats, from the abyss and deep sea to the air [8]. Measurements collected from the literature suggests that the IOP increased with the evolution of terrestrial vertebrates. The estimated IOP was indeed found to be smaller in amphibians and reptiles as compared to birds and mammals (Fig 10). The analysis carried out here shows that this rise is only weakly related to changes in the average body mass of animals. It could be a consequence of the transition from lens-based to cornea-based optics, which accompanied the evolution of terrestrial life and is likely to have been associated with a weakening of the power of the lens as compared to the cornea [6]. This transition may have necessitated an overall increase in the radius of curvature of the cornea (scaling Eq 5), which from our analysis would explain this increase in the average IOP.

### Specialisation and habitat/ecosystem

The evolution of the vertebrate eye has occurred along separate lines and has been largely determined by the ecosystems in which animals have developed. The eyes of amphibians share a number of characteristics with fish eyes, but also show many terrestrial adaptations. The eyes of reptiles and birds are completely adapted to aerial vision. The mammalian eye has evolved from a primitive reptilian source and adapted itself to almost every ecosystem, including a return to aquatic vision [6, 8]. For instance, specific adjustments of the eye for water and air vision have been found in penguins [148–150]. This evolution along separate lines is somehow visible when examining the IOP within each animal group. The IOP of aquatic birds and mammals is for instance closer to the IOP of respectively non-aquatic birds and mammals than it is to fish. This points towards an adaptation of the bird and mammalian eye to aquatic ecosystems while preserving some of their respective features. It is also in support of the idea that an evolution along separate lines produced different eye structures suitable to the same ecosystem or habitat [6, 8].

**Fig 11:**
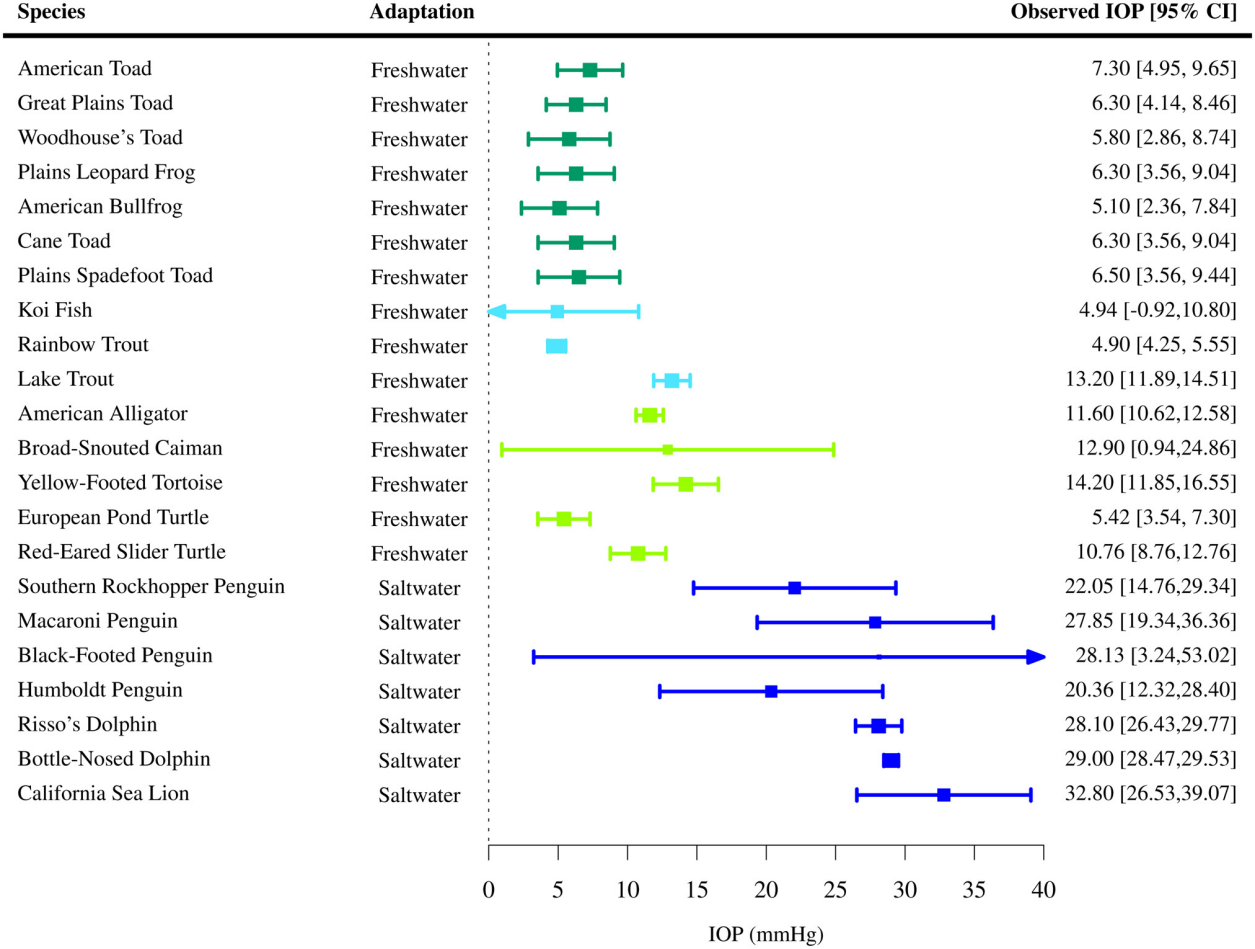
Observed IOP in vertebrates adapted to aquatic vision. The IOP appears to be lower in aquatic animals adapted to freshwater as compared to seawater. However, the group of animals adapted to saltwater is here limited to mammals. Among all the species adapted to fresh and saltwater the residual heterogeneity is I^2^ = 90.09%. Including saltwater and freshwater adaptations to the model as moderators accounts for 88.95% of the heterogeneity in effect.

The high heterogeneity in IOP observed among vertebrates suggests that it is weakly dependent of the environment in which each species has evolved. This is explained by the fact that the evolutionary constraint, namely that the IOP is required to overcome deformation due to head movement, is essentially the same in every ecosystem or habitat. However, specialisation of the visual function in certain ecosystems may have necessitated further adaptation of the IOP. The data collected in the present analysis suggests that the IOP is for instance higher in animals adapted to saltwater as compared to animals adapted to freshwater (Fig 11). The eye of seawater animals may have to withstand higher changes in pressure resulting from deep diving, which would in the light of the present analysis explain why their IOP is comparatively higher. This difference could also be caused by losses of water ocurring on the surface of the cornea of sea animals [143], which could be compensated for by increasing the rate of formation of aqueous humour. From Goldmann’s Eq (1), at equivalent episcleral venous pressure and bulk permeability, this would require an increase of the IOP.

The variations in IOP within each vertebrate group may also be partly explained by the existence of visual mechanisms (specific to some animals) that necessitate alterations of the IOP. In cetaceans, it has been hypothesised that accommodation is achieved by axial displacement of the lens resulting from changes in the IOP [151], which may explain the comparatively larger IOP observed in aquatic mammals as compared to other mammals. In arctic reindeers, the IOP, which was found to be significantly larger in winter than in summer, plays an important role in the changes in visual function associated with the adaptation to continuous summer light and continuous winter darkness [152]. The present work will hopefully pave the way to further analysis of these types of adaptations.

### Relevance for other sensory organs

The evolutionary constraints on the eye are likely to be similar to that of other closely related sensory organs, such as the semicircular canals of the inner ear. The inner ear senses unsteady movement by the deflection of hairs on the inside of the semicircular canals. In the same way that IOP is independent of the mass of the animal, the size of the semicircular canals of jawed vertebrates or gnathostomes are also independent of mass [35]. Various complex constraints and models have been proposed in the past to estimate the size of the inner ear and relative independence to *M*. The simplest explanation is that it senses the movement of velocity of the head which scales as *L*/*T*, which as was shown in the present work is weakly dependent on the mass of the animal.

## Conclusion

Using scaling and allometric analyses, the present work has highlighted fundamental evolutionary constraints in the development of the vertebrate eye, and further characterised the interdependence between the rate of formation of the aqueous humour, the metabolic requirements of the dioptric apparatus and the IOP. Importantly, the present study shows that animals need to be carefully selected when developing animal models for eye pathologies such as glaucoma. Even though the IOP lies within a range similar to all vertebrates, the rate of formation of aqueous humour is specific to each animal and changes between species. Given the interdependence between IOP and aqueous flow rate, it is essential to develop animal models having physiological characteristics similar to humans.

## Supporting Information

S1 Table Mean IOP, standard deviation and typical body mass of amphibians extracted through the systematic review. (PDF)

S2 Table Mean IOP, standard deviation and typical body mass of birds extracted through the systematic review. (PDF)

S3 Table Mean IOP, standard deviation and typical body mass of fish extracted through the systematic review. (PDF)

S4 Table Mean IOP, standard deviation and typical body mass of mammals extracted through the systematic review. (PDF)

S5 Table Mean IOP, standard deviation and typical body mass of reptiles extracted through the systematic review. (PDF)

S1 File Raw IOP Data. This file contains the mean IOP, standard deviation, sample size and type of tonometry used for the studies that satisfied the inclusion criteria.
(PDF)
