# Supplementary material for: Allometry and Scaling of the Intraocular Pressure and Aqueous Humour Flow Rate in Vertebrate Eyes
Source: PLoS One. 2016 Mar 18;11(3):e0151490. doi: 10.1371/journal.pone.0151490 (PMC4798774; doi:10.1371/journal.pone.0151490)
Supplement: S1 Table — (PDF) [file pone.0151490.s001.pdf]

Mean IOP, standard deviation and typical body mass of amphibians extracted through the systematic review.

Species	Common name	Sources	Sample size (eyes)	Mean IOP (mmHg)	Standard Deviation (mmHg)	Typical Body Mass (kg)
Anaxyrus americanus	**American Toad**	[50]	70	7.3	1.2	21.5×10^{-3}
Anaxyrus cognatus	**Great Plains Toad**	[50]	68	6.3	1.1	65×10^{-3}
Anaxyrus woodhouseii	**Woodhouse's Toad**	[50]	36	5.8	1.5	70.5×10^{-3}
Lithobates blairi	**Plains Leopard Frog**	[50]	66	6.3	1.4	32.5×10^{-3}
Lithobates catesbeianus	**American Bullfrog**	[50]	12	5.1	1.4	107.5×10^{-3}
Rhinella marina	**Cane Toad**	[50]	8	6.3	1.4	317.5×10^{-3}
Spea bombifrons	**Plains Spadefoot Toad**	[50]	60	6.5	1.5	21×10^{-3}
