# Supplementary material for: Allometry and Scaling of the Intraocular Pressure and Aqueous Humour Flow Rate in Vertebrate Eyes
Source: PLoS One. 2016 Mar 18;11(3):e0151490. doi: 10.1371/journal.pone.0151490 (PMC4798774; doi:10.1371/journal.pone.0151490)
Supplement: S2 Table — (PDF) [file pone.0151490.s002.pdf]

Mean IOP, standard deviation and typical body mass of birds extracted through the systematic review.

Species	Common name	Sources	Sample size (eyes)	Mean IOP (mmHg)	Standard Deviation (mmHg)	Typical Body Mass (kg)
Accipiter cooperii	**Cooper's Hawk**	[51]	6	13.35	0.67	0.46
Accipiter gentilis	**Northern Hawk**	[40]	18	21.2	2.4	1
Accipiter nisus	**Eurasian Sparrowhawk**	[40]	26	16.0	2.48	0.23
Aquila chrysaetos	**Golden Eagle**	[52]	14	21.5	3.0	5
Asio otus	**Long-Eared Owl**	[40, 53]	21	8.75	3.04	0.31
Athene noctua	**Little Owl**	[53, 54]	44	10.05	1.56	0.18
Bubo Bubo	**Eurasian Eagle Owl**	[55]	20	9.9	0.30	3
Bubo Bubo ascalaphus	**Pharaoh Eagle Owl**	[53]	2	7.8	3.5	3
Bubo Bubo interpositus	**Aharoni's Eagle Owl**	[53]	4	15.4	4.1	3
Bubo virginianus	**Great-Horned Owl**	[51, 52]	25	10.13	3.38	2
Buteo Buteo	**Common Buzzard**	[40, 53, 54]	116	23.71	12.0	0.9
Buteo buteo vulpinus	**Steppe Buzzard**	[53]	2	25.0	1.6	0.9
Buteo jamaicensis	**Red-Tailed Hawk**	[51, 52]	64	20.15	7.52	1
Buteo rufinus	**Long-Legged Buzzard**	[53]	4	13.3	4.0	1.2
Buteo swainsoni	**Swainson's Hawk**	[52]	12	20.8	2.3	0.98
Carthartes aura	**Turkey Vulture**	[51]	6	13.35	1.78	1.55
Circaetus aeruginosus	**Short-Toed Snake Eagle**	[53]	4	13.6	0.8	1.75
Circus gallicus	**Western March Harrier**	[53]	4	18.6	2.2	0.53
Circus pygargus	**Montagu's Harrier**	[53]	4	13.4	1.1	0.31
Eudyptes chrysocome	**Southern Rockhopper Penguin**	[56]	32	22.05	3.72	2.7
Eudyptes chrysolophus	**Macaroni Penguin**	[56]	50	27.85	4.34	5.5

Species	Common name	Sources	Sample size (eyes)	Mean IOP (mmHg)	Standard Deviation (mmHg)	Typical Body Mass (kg)
Falco pelegrinoides	**Barbary Falcon**	[53]	2	9.2	1.8	0.54
Falco naumanni	**Lesser Kestrel**	[53]	2	13.7	1.0	0.15
Falco peregrinus	**Peregrine Falcon**	[40]	4	15.3	6.1	0.89
Falco sparverius	**American Kestrel**	[51]	8	7.65	8.5	0.17
Falco tinnunculus	**Common Kestrel**	[40, 53, 54]	95	10.42	3.77	0.17
Haliaeetus albicilla	**White-Tailed Sea Eagle**	[40]	25	27.8	5.7	5
Haliaeetus albicilla	**Bald Eagle**	[52, 57]	37	21.38	0.38	4.65
Megascops asio	**Eastern Screech Owl**	[51, 58]	46	11.12	1.25	0.16
Milvus migrans	**Black Kite**	[53]	10	17.1	7.2	0.79
Milvus milvus	**Red Kite**	[40]	6	12.7	6.5	1.05
Otus scops	**Scops Owl**	[53]	23	14.5	3.9	0.05
Pernis apivorus	**European Honey Buzzard**	[53]	18	14.4	2.8	0.75
Phoenicopterus ruber	**American Flamingo**	[59, 60]	74	12.64	4.52	3.1
Poicephalus rufiventris	**Red-Bellied Parrot**	[61]	2	11.5	0.71	0.14
Rhea americana	**Great Rhea**	[62]	2	11.5	0.71	24
Spheniscus dermersus	**Black-Footed Penguin**	[63, 64]	70	28.76	12.70	2.82
Spheniscus humboldti	**Humboldt Penguin**	[65]	48	20.36	4.1	4.75
Strix aluco	**Eurasian Tawny Owl**	[40, 54]	52	11.18	0.05	0.6
Strix varia	**Barred Owl**	[51]	3	10.0	2.3	0.78
Struthio camelus	**Ostrich**	[66]	40	18.3	3.5	103
Tyto alba	**Barn Owl**	[40, 53]	33	17.21	7.12	0.41
