# Supplementary material for: Allometry and Scaling of the Intraocular Pressure and Aqueous Humour Flow Rate in Vertebrate Eyes
Source: PLoS One. 2016 Mar 18;11(3):e0151490. doi: 10.1371/journal.pone.0151490 (PMC4798774; doi:10.1371/journal.pone.0151490)
Supplement: S3 Table — (PDF) [file pone.0151490.s003.pdf]

Mean IOP, standard deviation and typical body mass of fish extracted through the systematic review.

Species	Common name	Sources	Sample size (eyes)	Mean IOP (mmHg)	Standard Deviation (mmHg)	Typical Body Mass (kg)
Cyprinus carpio	**Koi Fish**	[67]	18	4.94	2.99	0.75
Salvelinus gairdneri	**Rainbow Trout**	[68]	54	4.9	0.33	1.4
Salvelinus namaycush	**Lake Trout**	[68]	61	13.2	0.67	0.150
