# Supplementary material for: Allometry and Scaling of the Intraocular Pressure and Aqueous Humour Flow Rate in Vertebrate Eyes
Source: PLoS One. 2016 Mar 18;11(3):e0151490. doi: 10.1371/journal.pone.0151490 (PMC4798774; doi:10.1371/journal.pone.0151490)
Supplement: S4 Table — (PDF) [file pone.0151490.s004.pdf]

Mean IOP, standard deviation and typical body mass of mammals extracted through the systematic review.

Species	Common name	Sources	Sample size (eyes)	Mean IOP (mmHg)	Standard Deviation (mmHg)	Typical Body Mass (kg)
Adax nasomaculatus	**Addax Antelope**	[69]	36	11.2	3.2	85.6
Aepyceros melampus	**Impala**	[69]	8	8.0	1.2	53.5
Arctocephalus pusillus pusillus	**South African Fur Seal**	[70]	2	31.5	0.7	210
Bos taurus taurus	**Cattle**	[71, 72]	138	25.97	14.30	900
Canis lupus familiaris	**Dog (Beagle)**	[73]	4	12.8	2.1	10
Capra hicus	**Pigmy Goat**	[74]	20	10.17	0.30	35
Capra ibex nubiana	**Nubian Ibex**	[75]	24	17.95	4.78	39.2
Castor canadensis	**Canadian Beaver**	[76]	32	17.95	4.64	14.5
Cebus abella	**Capuchin Monkey**	[77]	30	18.4	3.8	3.5
Ceratotherium simum	**Wide-Lipped Rhinoceros**	[69]	22	32.1	10.4	2055
Chinchilla lanigera	**Chinchilla**	[78, 79]	142	17.87	6.26	0.5
Connochaetes gnou	**Wide-tailed Wildebeest**	[69]	20	15.5	3.7	121
Dama mesopotamica	**Asian Fallow Deer**	[80]	15	11.9	3.3	65
Equus burchelli	**Grant Zebra**	[75]	26	26.26	1.03	138
Equus ferus caballus	**Horse**	[72]	20	24.25	0.93	500
Felis catus	**Cat**	[81–84]	97	19.68	4.33	3.0
Gazella thomsoni	**Thomson's Gazelle**	[85]	22	7.6	1.6	22
Gorilla gorilla gorilla	**Western Lowland Gorilla**	[86]	10	12.0	4.3	125
Grampus griseus	**Risso's Dolphin**	[87]	2	28.1	0.85	400
Hemiechinus auritus	**Long-Eared Hedgehog**	[88]	28	20.1	4.0	0.38

Species	Common name	Sources	Sample size (eyes)	Mean IOP (mmHg)	Standard Deviation (mmHg)	Typical Body Mass (kg)
Homo sapiens sapiens	**Human**	[89, 90]	154	15.48	1.92	65
Hydrochaeris hydrochaeris	**Capybara**	[91]	44	16.47	4.28	50
Lama glama	**Llama**	[92]	32	13.10	0.35	165
Macaca fascicularis	**Cynomolgus Monkey**	[93, 94]	20	19.48	5.83	5
Macaca Mulatta	**Rhesus Macaque**	[95–97]	370	16.83	3.63	6.5
Macropus fuliginosus	**Western Gray Kangaroo**	[98]	16	14.04	3.47	41
Macropus rufus	**Red Kangaroo**	[41]	20	17.45	7.23	51
Mazama gouazoubira	**Brown Brocket Deer**	[99]	18	15.3	3.1	18
Mus musculus	**Mouse (Swiss White)**	[100–102]	34	15.84	1.65	0.03
Mustela putorius furo	**Ferret**	[103]	30	14.5	3.27	1.45
Orycteropus afer	**Aardvark**	[104]	2	15.5	2.12	52.5
Oryctolagus cuniculus cuniculus	**Rabbit (New Zealand White)**	[105–109]	125	19.71	3.17	3
Oryx dammah	**Scimitar-horned Oryx**	[69]	14	15.8	1.5	111.4
Oryx leucoryx	**Arabian Oryx**	[75]	15	15.4	26.67	132
Ovis aries	**Sheep**	[110]	10	14.57	0.43	35
Panthera leo	**Lion**	[111]	44	22.9	0.89	125
Phascolarctos cinereus	**Koala**	[112]	40	24.2	6.45	9.5
Pongo pygmaeus	**Orangutan**	[113]	2	13.5	0.71	50
Pteropus hypomelanus	**Island Flying Fox**	[114]	20	14.78	0.01	0.194
Pteropus pumilus	**Little Golden-Mantled Flying Fox**	[114]	20	19.07	0.30	0.5
Pteropus vampyrus	**Malayan Flying Fox**	[114]	20	16.2	0.55	0.9
Rattus norvegicus	**Rat (Lewis)**	[115, 116]	255	17.29	7.37	0.3

**Species**	**Common name**	**Sources**	**Sample size (eyes)**	**Mean IOP (mmHg)**	**Standard Deviation (mmHg)**	**Typical Body Mass (kg)**
Rhinoceros unicornis	**One-Horned Rhinoceros**	[117]	10	31.2	6.62	2055
Rusa unicolor	**Sambar Deer**	[42]	40	11.4	2.8	102
Sus scrofa domesticus	**Domestic Pig**	[118]	14	14.1	2.2	20
Tursiops truncatus	**Bottle-Nosed Dolphin**	[87]	4	29	0.27	400
Vicugna pacos	**Alpaca**	[92]	36	14.85	0.45	75
Zalophus californianus	**California Sea Lion**	[119]	39	32.8	3.2	250
