# Supplementary material for: Allometry and Scaling of the Intraocular Pressure and Aqueous Humour Flow Rate in Vertebrate Eyes
Source: PLoS One. 2016 Mar 18;11(3):e0151490. doi: 10.1371/journal.pone.0151490 (PMC4798774; doi:10.1371/journal.pone.0151490)
Supplement: S5 Table — (PDF) [file pone.0151490.s005.pdf]

Mean IOP, standard deviation and typical body mass of reptiles extracted through the systematic review.

Species	Common name	Sources	Sample size (eyes)	Mean IOP (mmHg)	Standard Deviation (mmHg)	Typical Body Mass (kg)
Alligator mississippiensis	**American Alligator**	[43]	16	11.6	0.5	300
Caiman latirostris	**Broad-Snouted Caiman**	[120]	28	12.9	6.1	45
Chelonoidis carbonaria	**Red-footed Tortoise**	[121]	50	15.3	8.81	
Chelonoidis denticulata	**Yellow-footed Tortoise**	[122]	30	14.2	1.2	15
Cyclura cychlura Cyclura	**Andros Island Iguana**	[123]	104	4.89	1.73	2.5
Emys Orbicularis	**European Pond Turtle**	[124]	44	5.42	0.96	0.33
Terrapene carolina major	**Gulf Coast Box Turtle**	[125]	138	6.7	1.4	0.72
Terrapene carolina triunguis	**Three-toed Box Turtle**	[125]	48	8.3	1.5	0.41
Testudo Hermanni	**Hermann's Tortoise**	[126]	52	15.74	0.2	3.5
Trachemys scripta elegans	**Red-Eared Slider Turtle**	[127]	34	10.76	1.02	0.77
