# Supplementary material for: Allometry and Scaling of the Intraocular Pressure and Aqueous Humour Flow Rate in Vertebrate Eyes
Source: PLoS One. 2016 Mar 18;11(3):e0151490. doi: 10.1371/journal.pone.0151490 (PMC4798774; doi:10.1371/journal.pone.0151490)
Supplement: S1 File — This file contains the mean IOP, standard deviation, sample size and type of tonometry used for the studies that satisfied the inclusion criteria. (PDF) [file pone.0151490.s006.pdf]

Table A. Amphibians

Species	Common Name	Sample Size (eyes)	Mean (mmHg)	Standard Deviation (mmHg)	Method[1]	Weight (if indicated) (kg)	Source
Anaxyrus americanus	**American Toad**	70	7.3	1.2	TonoVet (P)	0.004-0.039	[50]
Anaxyrus cognatus	**Great Plains Toad**	68	6.3	1.1	TonoVet (P)	0.037-0.093	[50]
Anaxyrus woodhouseii	**Woodhouse's Toad**	36	5.8	1.5	TonoVet (P)	0.042-0.099	[50]
Lithobates blairi	**Plains Leopard Frog**	66	6.3	1.4	TonoVet (P)	0.006-0.059	[50]
Lithobates catesbeianus	**American Bullfrog**	12	5.1	1.4	TonoVet (P)	0.016-0.199	[50]
Rhinella marina	**Cane Toad**	8	6.3	1.4	TonoVet (P)	0.235-0.400	[50]
Spe bombifrons	**Plains Spadefoot Toad**	60	6.5	1.5	TonoVet (P)	0.005-0.037	[50]

[1] See Table F for types of tonometry and manufacturers.

Table B. Birds

Species	Common Name	Sample Size (eyes)	Mean (mmHg)	Standard Deviation (mmHg)	Method	Weight (if indicated) (kg)	Source
Accipiter cooperii	**Cooper's Hawk**	6	16.0	1.8	Tono-Pen XL	*n.i.*	[51]
			10.7	1.4	TonoVet (P)		
Accipiter gentilis	**Northern Goshawk**	18	21.2	2.4	TonoVet (D)	*n.i.*	[40]
Accipiter nisus	**Eurasian Sparrowhawk**	26	16.0	2.8	TonoVet (D)	*n.i.*	[40]
Aquila chrysaetos	**Golden Eagle**	14	21.5	3.0	Tono-Pen XL	*n.i.*	[52]
Asio otus	**Long-Eared Owl**	17	8.5	3.0	TonoVet (D)	*n.i.*	[40]
		4	9.8	1.2	Tono-Pen XL	*n.i.*	[53]
Athene noctua	**Little Owl**	4	12.3	2.5	Tono-Pen XL	*n.i.*	[53]
		40	9.83	3.41	Tono-Pen XL		[54]
Bubo bubo	**Eurasian Eagle Owl**	20	9.35	1.81	Tono-Pen XL	*n.i.*	[55]
		20	10.45	1.64	TonoVet		
Bubo bubo ascalaphus	**Pharaoh Eagle Owl**	2	7.8	3.5	Tono-Pen XL	*n.i.*	[53]
Bubo bubo interpositus	**Aharoni's Eagle Owl**	4	15.4	4.1	Tono-Pen XL	*n.i.*	[53]
Bubo virginianus	**Great-Horned Owl**	15	9.9	2.4	Tono-Pen XL	*n.i.*	[51]
			9.9	2.2	TonoVet (P)		
		10	10.8	3.6	Tono-Pen XL	*n.i.*	[52]

Buteo buteo	**Common Buzzard**	56	29.9	6.1	TonoVet (D)	*n.i.*	[40]
		20	19.4	3.9	Tono-Pen XL		[53]
		40	17.2	3.53	Tono-Pen XL		[54]
Buteo buteo vulpinus	**Steppe Buzzard**	2	25.0	1.6	Tono-Pen XL	*n.i.*	[53]
Buteo jamaicensis	**Red-Tailed Hawk**	44	20.3	2.8	Tono-Pen XL	*n.i.*	[51]
			19.8	4.9	TonoVet (P)		
		20	20.6	3.4	Tono-Pen XL	*n.i.*	[52]
Buteo rufinus	**Long-Legged Buzzard**	4	13.3	4	Tono-Pen XL	*n.i.*	[53]
Buteo swainsoni	**Swainson's Hawk**	12	20.8	2.3	Tono-Pen XL	*n.i.*	[52]
Carthartes aura	**Turkey vulture**	6	15.0	2.1	Tono-Pen XL	*n.i.*	[51]
			11.7	1.0	TonoVet (P)		
Circaetus aeruginosus	**Short-Toed Snake Eagle**	4	13.6	0.8	Tono-Pen XL	*n.i.*	[53]
Circus gallicus	**Western March Harrier**	4	18.6	2.2	Tono-Pen XL	*n.i.*	[53]
Circus pygargus	**Montagu's Harrier**	4	13.4	1.1	Tono-Pen XL	*n.i.*	[53]
Eudyptes chrysocome	**Southern Rockhopper Penguin**	32	24.1	5.09	TonoVet (D)	*n.i.*	[56]
			20.0	5.77	Tono-Pen XL		
Eudyptes chrysolophus	**Macaroni Penguin**	50	31.6 (healthy)	6.9 (healthy)	TonoVet (D)	*n.i.*	[56]
			24.1(healthy)	7.5 (healthy)	Tono-Pen XL		
Falco pelegrinoides	**Barbary Falcon**	2	9.2	1.8	Tono-Pen XL	*n.i.*	[53]
Falco naumanni	**Lesser Kestrel**	2	13.7	1.0	Tono-Pen XL	*n.i.*	[53]
Falco peregrinus	**Peregrine Falcon**	4	15.3	6.1	TonoVet (D)	*n.i.*	[40]
Falco sparverius	**American Kestrel**	8	8.5	4.4	Tono-Pen XL	*n.i.*	[51]
			6.8	1.7	TonoVet (P)		

Falco tinnunculus	**Common Kestrel**	19	11.6	2.7	TonoVet (D)	*n.i.*	[40]
		36	11.9	3.3	Tono-Vet XL	*n.i.*	[53]
		40	8.53	1.59	Tono-Pen XL	*n.i.*	[54]
Haliaeetus albicilla	**White-Tailed Sea Eagle**	25	27.8	5.7	TonoVet (D)	*n.i.*	[40]
Haliaeetus leucocephalus	**Bald Eagle**	5	20.6	2.0	Tono-Pen XL	*n.i.*	[57]
		32	21.5	1.7	Tono-Pen	*n.i.*	[52]
Megascops asio	**Eastern Screech Owl**	4	9.3	2.6	Tono-Pen XL	*n.i.*	[58]
			6.3	1.3	TonoVet (P)		
		42	9	1.8	Tono-Vet (P)	0.1623 (0.018.1 SD) (0.1314 – 0.1935)	[51]
			14	2.4	Tono-Vet (D)		
			11	1.9	TonoPen XL		
Milvus migrans	**Black Kite**	10	17.1	7.2	Tono-Vet XL	*n.i.*	[53]
Milvus milvus	**Red Kite**	6	12.7	6.5	TonoVet (D)	*n.i.*	[40]
Otus scops	**Scops Owl**	23	14.5	3.9	Tono-Vet XL	*n.i.*	[53]
Pernis apivorus	**European Honey Buzzard**	18	14.4	2.8	Tono-Vet XL	*n.i.*	[53]
Phoenicopterus Ruber	**American Flamingo**	56	11.0 (upright) 14.3 (feeding)	1.6 (upright) 2.2 (feeding)	TonoVet	*n.i.*	[60]
		16	16.1	4.2	TonoPen XL	3.1 (0.48 SD) (2.2 – 4.4)	[59]
		18	9.5	1.7	TonoVet		
Poicephalus rufiventris	**Red-Bellied Parrot**	1	11 (left) 12 (right)		*n.i.*	*n.i.*	[61]
Rhea Americana	**Great Rhea**	1	12 (left) 11 (right)		TonoPen XL	*n.i.*	[62]

Spheniscus dermersus	**Black-Footed Penguin**	36	31.77	3.3	TonoVet	2.816 (SD not reported)	[63]
		34	28.13 (left)<br>30.41 (right)	6.84 (left)<br>4.27 (right)	TonoVet (D)	*n.i.*	[64]
			25.05 (left)<br>25.06 (right)	5.56 (left)<br>4.35 (right)	TonoVet (H)		
Spheniscus humboldti	**Humboldt Penguin**	48	20.36	4.1	Tono-Pen XL	*n.i.*	[65]
Strix aluco	**Eurasian Tawny Owl**	12	11.1	3.1	TonoVet (D)	*n.i.*	[40]
		40	11.21	3.12	Tono-Pen XL	*n.i.*	[54]
Strix varia	**Barred Owl**	3	11.7	3.8	Tono-Pen XL	*n.i.*	[51]
			8.3	3.2	TonoVet (P)		
Struthio camelus	**Ostrich**	40	18.3	3.5	Tono-Pen	*n.i.*	[66]
Tyto alba	**Barn Owl**	4	11.5	4.7	TonoVet (D)	*n.i.*	[40]
		29	18.0	6.6	Tono-Pen XL	*n.i.*	[53]

Table C. Fish

Species	Common Name	Sample Size (eyes)	Mean (mmHg)	Standard Deviation (mmHg)	Method	Weight (if indicated) (kg)	Source
Cyprinus carpio	**Koi Fish**	18	4.944	2.99	TonoVet (D)	*n.i.*	[67]
Salvelinus gairdneri	**Rainbow Trout**	54	4.9	0.33	Manometer	*n.i.*	[68]
Salvelinus namaycush	**Lake Trout**	61	13.2	0.67	Manometer	0.150 (0.0042 SD)	[68]

Table D. Mammals

Species	Common Name	Sample Size (eyes)	Mean (mmHg)	Standard Deviation (mmHg)	Method	Weight (if indicated) (kg)	Source
Adax nasomaculatus	**Addax Antelope**	36	11.2	3.2	Tono-Pen XL	85.6 (35.6 SD)	[69]
Aepyceros melampus	**Impala**	8	8.0	1.2	Tono-Pen XL	53.5 (28.7 SD)	[69]
Arctocephalus pusillus pusillus	**South African Seal**	2	31.5	0.7	Tono-Pen	*n.i.*	[70]
Bos taurus taurus	**Cattle**	20	18.8	1.7	Perkins	*n.i.*	[72]
			19.7	1.2	Manometer		
		64	27.5	4.8	Mackay-Marg	*n.i.*	[71]
		54	28.2	4.6	Mackay-Marg	*n.i.*	
			26.9	6.7	Tono-Pen XL		
Canis lupus familiaris	**Dog (Beagle)**	4	12.8	2.1	Langham	*n.i.*	[73]
Capra hicus	**Pigmy Goat**	20	11.8	1.5	TonoVet (D)	33.7 (SD *n.i.*)	[74]
			7.9	1.7	Tono-Vet (P)		
			10.8	1.7	Tono-Pen XL		
Capra ibex nubiana	**Nubian Ibex**	24	17.95	4.78	Schiotz	39.1 (8.3 SD)	[75]
Castor canadensis	**Canadian Beaver**	32	18.79 (left)<br>17.11 (right)	5.63<br>6.39	Tono-Pen XL	7-20	[76]
Cebus abella	**Capuchin Monkey**	30	18.4	3.8	Tono-Pen XL	2-5	[77]
Ceratotherium simum	**Wide-Lipped Rhinoceros**	22	32.1	10.4	Tono-Pen XL	2055 (235 SD)	[69]
Chinchilla lanigera	**Chinchilla**	114	17.71	4.17	Tono-Pen XL	0.57069 (0.10869 SD)	[78]
		28	18.5	5.75	Mackay-Marg	0.475-0.675	[79]

Connochaetes gnou	**Wide-Tailed Wildebeest**	20	15.5	3.7	Tono-Pen XL	121 (68.2 SD)	[69]
Dama mesopotamica	**Asian Fallow Deer**	15	11.9	3.3	Tono-Pen XL	*n.i.*	[80]
Equus burchelli	**Grant Zebra**	20	25.30	3.06	Schiotz	138 (24 SD)	[75]
		6	29.47	3.43	Tono-Pen XL		
Equus ferus caballus	**Horse**	20	25.1	2.9	Manometer	*n.i.*	[72]
			23.4	3.2	Perkins		
Felis catus	**Cat**	8	17	0.8	Schiotz	*n.i.*	[81]
		7	16	2.3			
		12	12.7	1.1	Pneumato-nometer	*n.i.*	[83]
		8	24.6	3.1	Pneumato-nometer	3-4	[82]
		8	24.3	3.6			
		8	20.9	3.0			
		8	20.8	3.1			
		6	23.2	3.4			
		6	24.5	1.2			
		7	21.4	1.1			
		7	20.4	2.0			
		6	17.4	1.5	Manometer	2.0-3.5	[84]
		6	16.7	1.6			
Gazella thomsoni	**Thompson's Gazelle**	22	7.6	1.6	Tono-Pen XL	22 (3.1 SD)	[85]
Gorilla gorilla gorilla	**Western Lowland Gorilla**	10	12.0	4.3	Schiotz	90.9 – 159	[86]
Grampus griseus	**Risso's Dolphin**	2	28.7 (left)<br>27.5 (right)	–	Tono-Pen	*n.i.*	[87]
Hemiechinus auritus	**Long-Eared Hedgehog**	28	20.1	4.0	Tono-Pen Vet	0.3785 (0.0924 SD)	[88]
Homo sapiens sapiens	**Human**	80	16.3	2.9	Goldmann	*n.i.*	[90]
		74	14.6	3.5	Goldmann	*n.i.*	[89]
Hydrochaeris hydrochaeris	**Capybara**	44	16.47	4.28	Tono-Pen XL	37 – 63	[91]

Lama glama	**Llama**	32	13.10	0.35		*n.i.*	[92]
Macaca fascicularis	**Cynomolgus Monkey**	8	19.3	0.8	Goldman	3-8	[94]
		12	19.6	3.5	Pneumato-nometer	*n.i.*	[93]
Macaca mulatta	**Rhesus Macaque**	11	16.1	0.9	Goldman	*n.i.*	[95]
		8	19.1	1.1			
		5	18.3	1.6			
		326	16.9	3.8	Applana-tion		[97]
		20	14.9	2.5	TonoPen XL		[96]
Macropus fuliginosus	**Western Grey Kangaroo**	16<br>12	10.69 (April 2008)<br>9.0 (May 2008)	3.59<br>*n.i.*	TonoVet	22 – 63	[98]
		16<br>12	17.38 (April 2008)<br>11.5 (May 2008)	4.44<br>*n.i.*	Tono-Pen XL		
Macropus rufus	**Red Kangaroo**	20	17.45	7.23	Tono-Pen XL	17 – 35 (female)<br>22 – 85 (male)	[41]
Mazama gouazoubira	**Brown Brocket Deer**	18	15.3	3.1	Tono-Pen XL	*n.i.*	[99]
Mus musculus	**Mouse (Swiss White)**	20	15.7	2.0	Manometer	*n.i.*	[101]
		6	16.5	0.6	Manometer	*n.i.*	[100]
		8	15.7	1.0	Manometer	*n.i.*	[102]
Mustela putorius furo	**Ferret**	30	14.5	3.27	Tono-Pen XL	*n.i.*	[103]
Orycteropus afer	**Aardvark**	2	17.0 (left)<br>14.0 (right)		Tono-Pen XL	57 – 59	[104]

Oryctolagus cuniculus	**Rabbit (New Zealand White)**	8	20.1	1.52	Pneumato-nometer	2-2 .5	[108]
		9	21.7	0.88			
		11	17.2	0.9			
		6	21.0	1.5 (SE)	Pneumato-nometer	2.5-4.0	[109]
		21	17.24	0.25 (SEM)	Pneumato-nometer	3-4	[107]
		10	20.1	0.8 (SEM)	Pneumato-nometer	3-4	[106]
		60	20.5	0.2	Pneumato-nometer	2.1-2.7	[105]
Oryx dammah	**Scimitar-Horned Oryx**	14	15.8	1.5	Tono-Pen XL	111.4 (63.2 SD)	[69]
Oryx leucoryx	**Arabian Oryx**	5	22.68	8.15	Schiotz	132 (16.4 SD)	[75]
		10	11.76	11.3.43	Tono-Pen XL		
Ovis aries	**Sheep**	10	15.20 (left)<br>14.70 (left)<br>12.70 (left)<br><br>16.2 (right)<br>14.70 (right)<br>13.90 (right)	1.06<br>1.15<br>1.09<br><br>1.41<br>1.34<br>0.84	Tono-Pen XL	35.73 (1.23 SD)	[110]
Panthera leo	**Lion**	44<br>(22M + 22W)	20.9 (female)<br>24.9 (male)	2.4<br>2.0	Schiotz	120 (20.3 SD)	[111]
Phascolarctos cinereus	**Koala**	40	24.2	6.45	Manometer	*n.i.*	[112]
Pongo pygmaeus	**Orangutan**	2	13 (left)<br>14 (right)		Tono-Pen XL	50	[113]
Pteropus hypomelanus	**Island Flying Fox**	20	12.1 (upright)<br>17.45 (hanging)	1.10<br>1.11	TonoVet	0.425 – 0.571	[114]
Pteropus pumilus	**Little Golden-Mantled Flying Fox**	20	15.63 (upright)<br>22.5 (hanging)	1.01<br>1.27	TonoVet	0.194	[114]
Pteropus vamyprus	**Malayan Flying Fox**	20	14.2 (upright)<br>18.2 (hanging)	0.89<br>1.37	TonoVet	0.645 – 1.153	[114]
Rattus norvegicus	**Rat (Lewis)**	26	17.25	1.8	Tono-Pen	0.2946 (SD 0.069)	[115]
		229	17.30	5.25	Pressure Transducer	0.15-0.2	[116]
Rhinoceros unicornis	**One-Horned Rhinoceros**	10	31.2	6.62	Tono-Pen	*n.i.*	[117]

Rusa unicolor	**Sambar Deer**	40	11.4	2.8	Tono-Pen XL	102.77 (3.35 SD)	[42]
Sus scrofa domesticus	**Domestic Pig**	14	14.1	2.2	*n.i.*	20	[118]
Tursiops truncatus	**Bottle-Nosed Dolphin**	4	33.4 (male)<br>24.6 (female)	2.4<br>2.3	Tono-Pen	*n.i.*	[87]
Vicugna pacos	**Alpaca**	36	14.85	0.45		*n.i.*	[92]
Zalphus californianus	**California Sea Lion**	39	32.8	3.2	TonoVet	*n.i.*	[119]

Table E. Reptiles

Species	Common Name	Sample Size (eyes)	Mean (mmHg)	Standard Deviation (mmHg)	Method	Weight (if indicated) (kg)	Source
Alligator missipensis	**American Alligator**	16	11.6	0.5	Applana-tion	*n.i.*	[43]
Caiman latirostris	**Broad-Snouted Caiman**	28	12.9	6.1	Tono-Pen XL	*n.i.*	[120]
Chelonoidis carbonaria	**Red-Footed Tortoise**	50	15.3	8.81	Tono-Pen	*n.i.*	[121]
Chelonoidis denticulate	**Yellow-Footed Tortoise**	30	14.2	1.2	Tono-Pen	*n.i.*	[122]
Cyclura cychlura cyclura	**Andros Island Iguana**	104	4.89	1.73	TonoVet (P)	2.4766 (1.663 SD)	[123]
Emys Orbicularis	**European Pond Turtle**	44	5.42	0.96	TonoVet (P)	0.39228 (0.0731 SD) (female)<br>0.25884 (0.073 SD) (male)	[124]
Terrapene carolina major	**Gulf Coast Box Turtle**	138	6.7	1.4	TonoVet (P)	0.7217 (0.1561 SD)	[125]
Terrapene carolina triunguis	**Three-Toed Box Turtle**	48	8.3	1.5	TonoVet (P)	0.4195 (0.1037 SD)	[125]
Testudo hermanni	**Hermann's Tortoise**	52	15.74	0.2	Rebound	0.255 – 2.310	[126]
Trachemys scripta elegans	**Red-Eared Slider Turtle**	34	10.2	0.66	TonoLab	0.770 (0.130 SD)	[127]
			11.32	1.57	TonoVet		

Table F. List of tonometers

Tonometer	Type of Tonometry	Manufacturer (if commercial tonometer)	Notes
Tono-Pen	Applanation	Reichert Technologies, Buffalo, NY, USA	–
Tono-Pen XL	Applanation	Reichert Technologies, Buffalo, NY, USA	–
Tono-Pen Vet	Applanation	Reichert Technologies, Buffalo, NY, USA	–
TonoVet	Rebound	Icare Finland Oy, Vanta, Finland	**Three calibrations:** (D) Dog (H). Horse (P). No specific species calibration
TonoLab	Rebound	Icare Finland Oy, Vanta, Finland	–
Perkins	Applanation	Haag-Streit, Harlow, UK	–
Mackay-Marg	Applanation and Indentation	–	–
Langham	Applanation	–	–
Schiotz	Indentation	–	–
Manometer	Pneumatonometer/ Pressure transducer	–	–
Goldman	Applanation	–	–
